# Near‐Infrared‐II Nanoparticles for Vascular Normalization Combined with Immune Checkpoint Blockade via Photodynamic Immunotherapy Inhibit Uveal Melanoma Growth and Metastasis

**DOI:** 10.1002/advs.202206932

**Published:** 2023-11-08

**Authors:** Xiaoqin Zheng, Yunyi Shi, Dongsheng Tang, Haihua Xiao, Kun Shang, Xuezhi Zhou, Gang Tan

**Affiliations:** ^1^ Department of Ophthalmology The First Affiliated Hospital Hengyang Medical School University of South China Hengyang Hunan 421001 P. R. China; ^2^ Beijing National Laboratory for Molecular Sciences State Key Laboratory of Polymer Physics and Chemistry Institute of Chemistry Chinese Academy of Sciences Beijing 100190 P. R. China; ^3^ University of Chinese Academy of Sciences Beijing 100049 P. R. China; ^4^ Institute of Medical Technology Peking University Health Science Center Beijing 100190 P. R. China; ^5^ Eye Center of Xiangya Hospital Central South University Changsha Hunan 410008 P. R. China

**Keywords:** abnormal vasculature, anti‐programmed death‐ligand 1, Photodynamic therapy, photodynamic‐immunotherapeutic, uveal melanoma

## Abstract

Photodynamic therapy (PDT) has been widely employed in tumor treatment due to its effectiveness. However, the tumor hypoxic microenvironment which is caused by abnormal vasculature severely limits the efficacy of PDT. Furthermore, the abnormal vasculature has been implicated in the failure of immunotherapy. In this study, a novel nanoparticle denoted as Combo‐NP is introduced, composed of a biodegradable NIR II fluorescent pseudo‐conjugate polymer featuring disulfide bonds within its main chain, designated as TPA‐BD, and the vascular inhibitor Lenvatinib. Combo‐NP exhibits dual functionality by not only inducing cytotoxic reactive oxygen species (ROS) to directly eliminate tumor cells but also eliciting immunogenic cell death (ICD). This ICD response, in turn, initiates a robust cascade of immune reactions, thereby augmenting the generation of cytotoxic T lymphocytes (CTLs). In addition, Combo‐NP addresses the issue of tumor hypoxia by normalizing the tumor vasculature. This normalization process enhances the efficacy of PDT while concurrently fostering increased CTLs infiltration within the tumor microenvironment. These synergistic effects synergize to potentiate the photodynamic‐immunotherapeutic properties of the nanoparticles. Furthermore, when combined with anti‐programmed death‐ligand 1 (PD‐L1), they showcase notable inhibitory effects on tumor metastasis. The findings in this study introduce an innovative nanomedicine strategy aimed at triggering systemic anti‐tumor immune responses for the treatment of Uveal melanoma.

## Graphical abstract

A nanoparticle (Combo‐NP) that contains biodegradable NIR‐II‐fluorescent pseudo conjugate polymer with disulfide bonds in the main chain (TPA‐BD) and a vascular inhibitor Lenvatinib (Len) is designed. Combo‐NP not only kill tumors by generating reactive oxygen species (ROS), but also trigger immunogenic cell death (ICD) initiating cascade immune responses leading to the production of cytotoxic T lymphocytes (CTLs). Moreover, Combo‐NP alleviate hypoxia by normalizing the tumor vessels, thereby improving the Photodynamic therapy (PDT)efficiency and increasing the infiltration of CTLs. These effects combine to amplify the photodynamic‐immunotherapy of the nanoparticles exhibiting promising inhibitory effects on tumor metastasis and inducing an abscopal immune response when combine with anti‐programmed death‐ligand 1 (PD‐L1) immunotherapy.

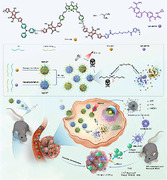

## 1 Introduction

Uveal melanoma (UM) is an invasive malignancy that arises from the melanocytes in the eye. Despite effective control of the primary tumor with radiation therapy or surgery, 50% of UM patients will develop metastatic disease. The overall survival rates of UM are poor, and to date, there is no standard approach for targeting primary UM and preventing further metastases simultaneously.^[^ ^1^ ^]^

Photodynamic therapy (PDT) is a promising treatment modality for intraocular tumors due to its effectiveness, non‐invasiveness, and safety.^[^ ^2^ ^]^ There are two ways to kill tumors during a typical PDT process. On the one hand, photosensitizers kill tumors directly by generating the cytotoxic reactive oxygen species (ROS) under light irradiation.^[^ ^3^ ^]^ On the other hand, PDT is proved to induce immunogenic cell death (ICD) stimulating tumor cells to release damage‐associated molecular patterns (DAMPs) and tumor‐associated antigens (TAAs),^[^ ^4^ ^]^ which trigger dendritic cells (DCs) maturation and amplify T lymphocyte infiltration, thus reversing “cold tumors” to “hot tumors” for immunotherapy.^[^ ^5^ ^]^ However, such photodynamic‐immunotherapy is extremely restricted by the major obstacle of tumor hypoxia induced by the abnormal vasculature especially for the solid tumors, which typically have heterogeneous cells growth that exceed their blood supply,^[^ ^6^ ^]^ thereby resulting in a very low oxygen concentration (hypoxic) in the tumor site and increasing the expression of hypoxia‐inducible factor and vascular endothelial growth factor (VEGF), resulting in an imbalance of pro‐angiogenic and anti‐angiogenic factors, ultimately to increase the expression of vessels with abnormal function and structure.^[^ ^7^ ^]^ These abnormal vascular networks are characterized by disorderly arrangement, immaturity, and high permeability. The abnormal vasculature in tumors results in hypoxia, which in turn reduces the generation of ROS.^[^ ^8^ ^]^ Additionally, the abnormal vasculature results in limited infiltration and activity of tumor‐specific cytotoxic T lymphocytes (CTLs) at the tumor site. Moreover, the abnormal vessels foster immune evasion by recruiting immunosuppressive immune cells into the tumor microenvironment (TME).^[^ ^9^ ^]^ Recent studies indicated that a multi‐targeted tyrosine kinase inhibitor Lenvatinib could inhibit vascular endothelial growth factor receptors (VEGFRs)1‐3, fibroblast factor receptors (FGFRs)1‐4, and platelet‐derived growth factor receptor (PDGFR) alpha, leading to normalization of blood vessels.^[^ ^10^ ^]^ Consequently, this could relieve hypoxia and increase immunoresponsiveness, thereby enhancing the effect of PDT.

**Scheme 1:**
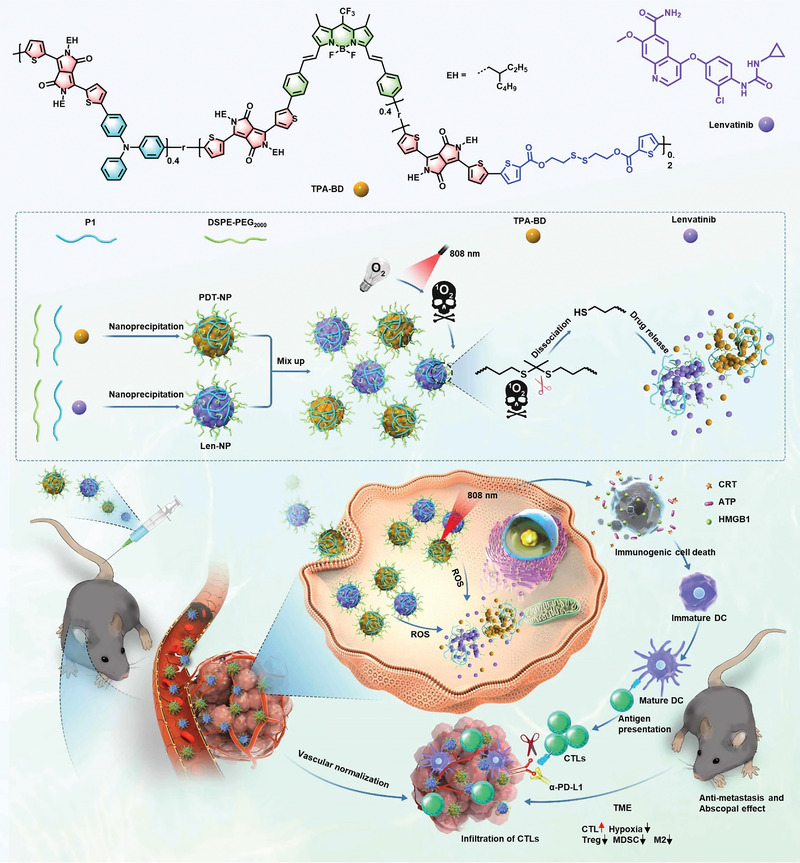
Schematic illustration of NIR‐II theranostic Combo‐NP for photodynamic‐immunotherapy via normalizing blood vessels. A NIR II fluorescent biodegradable pseudo conjugate polymer with disulfide bonds was designed (TPA‐BD). TPA‐BD was then adopted to deliver a multi‐targeted tyrosine kinase inhibitor, ie., Lenvatinib to normalize the tumor vessels, aiming to alleviate tumor hypoxia and improve the PDT efficiency. Subsequently, the normalized vasculature facilitated the infiltration and migration of CTLs into the tumor microenvironment while decreased the recruitment of immune‐suppressive cells (MDSC, Treg, M2). Furthermore, combination of α‐PD‐L1 mAb with Combo‐NP induced systemic antitumor‐mediated abscopal effect and inhibited cancer metastasis on mice cancer models.

Herein, a NIR II fluorescent biodegradable pseudo‐semiconducting polymer (TPA‐BD) with strong photodynamic effect was designed (**Scheme** **1**). TPA‐BD was characterized by having numerous tumor microenvironment‐responsive soft blocks with disulfide bonds and BODIPY units in the polymer main chain. Remarkably, TPA‐BD could be degraded rapidly in a sacrificial manner triggered by glutathione (GSH) in cancer cells.^[^ ^11^ ^]^ Thereafter, a ROS‐sensitive polymer (P1) was adopted together with DSPE‐PEG_2000_ to co‐assemble with TPA‐BD to form PDT‐NP. Meanwhile, P1 and DSPE‐PEG_2000_ encapsulated Lenvatinib into Len‐NP. Finally, PDT‐NP and Len‐NP were mixed in a certain ratio into Combo‐NP. Upon light irradiation, Combo‐NP could be dissociated by ROS to expose TPA‐BD and release Lenvatinib. TPA‐BD could further produce large amounts of ROS. On the one hand, TPA‐BD produced large amount of ROS to induce tumor cells to undergo ICD effect. Eventually, TPA‐BD was degraded by excessive intracellular GSH. Moreover, the released Lenvatinib enhanced the photodynamic‐immunotherapy by normalizing tumor vasculature, simultaneously enhanced CTLs infiltration, and decreased the accumulation of immunosuppressive cells at the tumor site. Furthermore, in our paper we demonstrated that the Combo‐NP enchanced the therapeutic efficacy of anti‐programmed death‐ligand 1 (PD‐L1) antibody through increasing the expression of PD‐L1 and converting the “cold tumor” into “hot tumor”, the combination of anti‐PD‐L1 antibody with Combo‐NP could activate the systemic immune response, demonstrating a promising inhibitory effect on tumor metastasis and abscopal immune response. In conclusion, Combo‐NP integrated NIR‐II fluorescence bioimaging, photodynamic‐immunotherapy and tumor vascular normalization. Furthermore, the combination of Combo‐NP and anti‐PD‐L1 was shown to provide an effective therapeutic strategy for UM patients and help to inhibit tumor growth and metastasis.

## 2 Results and Discussion

### 2.1 Synthesis of TPA‐BD and Characterization of Nanoparticles

**Figure 1:**
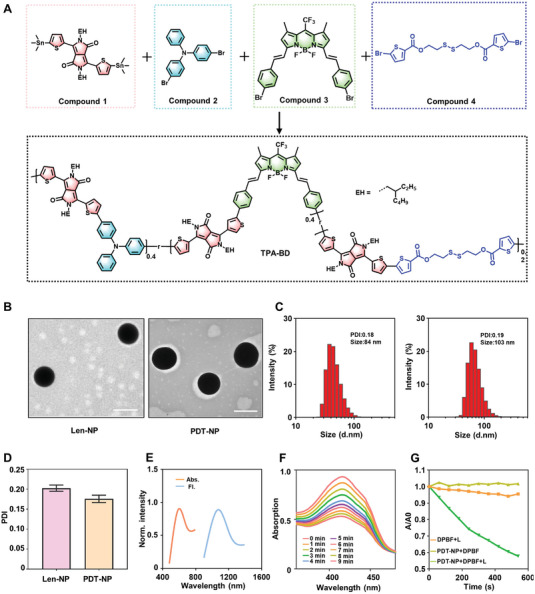
Synthesis of TPA‐BD and characterization of nanoparticles. A) Synthetic routes for NIR II fluorescent TPA‐BD. B) Characterization of Len‐NP and PDT‐NP by TEM. Scale bar = 100 nm. C) Diameter and D) polydispersity index of Len‐NP and PDT‐NP by DLS. E) Representative absorption and fluorescence emission spectra of PDT‐NP. F) Decay of DPBF absorption at 410 nm with increasing light irradiation time (0 min to 9 min) for PDT‐NP+DPBF+L. G) Decay of DPBF absorption at 410 nm for different samples (PDT‐NP+DPBF+L, PDT‐NP+DPBF, DPBF+L).

To obtain TPA‐BD, a Bodipy monomer (BD) was synthesized for polymerization (Figure S1, Supporting Information). BD was characterized by the presence of two end‐capped bromide elements (Figures S2 and S3, Supporting Information). Subsequently, BD (compound 3) could be polymerized with compound 1 (2,5‐bis(2‐ethylhexyl)−3,6‐bis(5‐(trimethylstannyl)thiophen‐2‐yl)−2,5‐dihydropyrrolo[3,4‐c]pyrrole‐1,4‐dione), compound 2 (4,4′‐Dibromotriphenylamine), and compound 4 (disulfanediylbis(ethane‐2,1‐diyl)bis(5‐bromothiophene‐2‐ carboxylate)) (Figure S4, Supporting Information) via a so‐called Stille reaction to give a pseudo‐semiconducting polymer (TPA‐BD) (**Figure** **1**A; Figure S5, Supporting Information). Notably, TPA‐BD was characterized as a non‐fully conjugated polymer due to the fact that there was a disulfide‐containing linkages in the polymer backbone, hence endowing it with biodegradability.^[^ ^11^ ^]^ Moreover, TPA‐BD could be excited by NIR light to generate ROS for PDT.

Subsequently, a biodegradable ROS‐sensitive polymer (P1) previously reported^[^ ^12^ ^]^ together with DSPE‐PEG_2000_ was adopted to co‐assemble with TPA‐BD to form nanoparticles via nanoprecipitation (PDT‐NP). Meanwhile, P1 and DSPE‐PEG_2000_ could encapsulate Lenvatinib (Len) into Len‐loaded nanoparticles (Len‐NP). Finally, Len‐NP and PDT‐NP were mixed in a certain ratio to the final Combo‐NP. Further study showed that both Len‐NP and PDT‐NP were at 100 nm in diameter by transmission electron microscopy (TEM) (Figure 1B). Further characterization by dynamic light scattering (DLS) indicated that the average size was 84 nm for Len‐NP, 103 nm for PDT‐NP, and 107 nm for Combo‐NP (Figure 1C; Figure S6A, Supporting Information), with a polydispersity index (PDI) at 0.19, 0.18, and 0.2, respectively (Figure 1D; Figure S6B, Supporting Information). Moreover, both Len‐NP and PDT‐NP were negatively charged with zeta‐potential at −13 mV and −17 mV, respectively (Figure S7, Supporting Information).

The photophysical properties of nanoparticles were then studied. Results indicated that PDT‐NP had a strong absorption peak centered at 604 nm. Moreover, there was strong fluorescence emission in the second NIR window (NIR II, 950–1700 nm) with a major peak centered at 1067 nm (Figure 1E). To verify the PDT effect of PDT‐NP, 1,3‐diphenylisobenzofuran (DPBF) was used as a probe to examine the ability of PDT‐NP+L to generate ROS. DPBF could produce more stable o‐dibenzoyl benzene with ^1^O_2,_^[^ ^13^ ^]^ and during this process the UV absorbance intensity of DPBF at 410 nm decreased with the generation of ^1^O_2_ (Figure 1F). The results showed that the absorbance intensity of DPBF decreased from 0.9162 to 0.5297 within 9 min, indicating that PDT‐NP+DPBF+L indeed generated ROS in a time‐dependent manner. However, the UV absorbance intensity at 410 nm of DPBF in the presence of PDT‐NP+DPBF and DPBF+L did not change significantly (Figure 1G), indicating PDT‐NP+DPBF (without light irradiation) and DPBF+L did not have PDT effect. The abovementioned ^1^O_2_ could further trigger the release of Lenvatinib. To prove this, high performance liquid chromatography (HPLC) was used to evaluate the release of Lenvatinib from Combo‐NP. The result showed that the Lenvatinib was released in a time‐dependent manner, with ≈80% of Lenvatnib being released at 48 h after light irradiation and almost no Lenvatinib being released in the dark (Figure S8A, Supporting Information). To further prove the ROS responsiveness of Combo‐NP, DLS was used to monitor the variation in diameters of Combo‐NP after light irradiation, as shown in Figure S8B (Supporting Information), the diameter of Combo‐NP became uneven, and the result of TEM further confirmed it (Figure S8C, Supporting Information).

### 2.2 Anticancer Activity of Combo‐NP+L In Vitro

**Figure 2:**
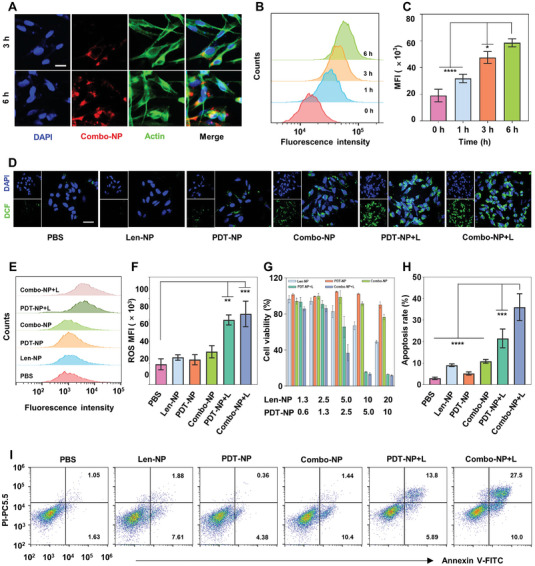
Combo‐NP exhibited PDT effect to show anticancer activity in vitro. A) Representative CLSM images of OCM1 cells treated with Combo‐NP at 3 h and 6 h, respectively. The blue fluorescence came from the cell nuclei stained with DAPI. The red fluorescence came from Combo‐NP and the green came from the cell skeleton stained by Alexa Fluor 488TM Phalloidin, respectively. Scale bar = 100 µm. B) Flow cytometric profiles and C) the corresponding quantification of intracellular uptake of Combo‐NP@Cy5.5 at 1 h, 3 h, and 6 h, respectively. D) ROS generation studied by DCFH‐DA in OCM1 cells via CLSM. Scale bar = 40 µm. E) Flow cytometric profiles and F) the corresponding quantification of ROS generation. G) Cell viabilities of OCM1 cells by MTT assay. H) The apoptosis rate in OCM1 cells and I) representative FCM images. *n* = 3. Data are presented as mean ± SD. Statistic significances between every two groups were calculated via one‐way ANOVA. * *p* < 0.05, **p < 0.01, *** *p* < 0.001, **** *p*<0.0001.

The internalization of Combo‐NP is the first step to exert a good anti‐cancer effect. To visualize the endocytosis of Combo‐NP, OCM1 cells were incubated with Combo‐NP at different times and the intracellular uptake was analyzed by Confocal laser microscope imaging (CLSM) via NIR II fluorescence (λ_ex_ = 808 nm). The results showed that the NIR‐II fluorescence was gradually intensified with increasing incubation time of Combo‐NP in OCM1 cells (**Figure** **2**A; Figure S9, Supporting Information). Subsequently, to quantify the cellular uptake of Combo‐NP, Cy5.5 was labeled with Combo‐NP (Combo‐NP@Cy5.5) and the endocytosis of Combo‐NP was further analyzed using flow cytometry (FCM) (Figure 2B). The results showed that the fluorescence intensity at 6 h was nearly twice as high as that at 1 h (Figure 2C). In conclusion, the above results demonstrated Combo‐NP could be internalized effectively by OCM1 and employed to monitor the internalization of nanoparticles by NIR‐II imaging. Further, the PDT effect of Combo‐NP was investigated. Here, dichlorofluorescein diacetate (DCFH‐DA) was used as a probe to detect ROS generation. DCFH‐DA itself does not emit fluorescence, but it can be hydrolyzed by intracellular esterase to DCFH, which can be further oxidized by ROS to DCF that emits a strong green fluorescence.^[^ ^14^ ^]^ The stronger the green fluorescence is, the more ROS generation is. The results showed that the OCM1 cells treated with PDT‐NP+L and Combo‐NP+L had stronger green fluorescence than those treated with PBS observed by CLSM (Figure 2D). Meanwhile, the ROS generation was quantified by FCM, and the results showed that the ROS generation of cells treated with Combo‐NP+L (65 778) was almost 3.5 times more than that of cells treated with PBS (18 939) (Figure 2E,F). The above study fully indicated a stronger ability of Combo‐NP+L to generate ROS. ROS can induce cytotoxic effects by damaging biological macromolecules (nucleic acids, proteins, lipids, *etc*.). First, the cytotoxicity of Combo‐NP+L on OCM1 was evaluated by MTT assay. The results showed that the viability of OCM1 cells remained >90% even at a PDT‐NP concentration up to 10 µg mL^−1^. However, PDT‐NP+L resulted in a cell inhibition at 87% on OCM1 cells at the same concentration of 10 µg mL^−1^, indicating that PDT‐NP had almost no toxicity without light irradiation. On the other hand, Combo‐NP+L showed a dramatic cell‐killing effect on OCM1 cells (63% cell inhibition), which was 1.9 times higher than that of PDT‐NP+L (34%) at a concentration of 2.5 µg mL^−1^ (Figure 2G). What's more, the enhanced potency of Combo‐NP+L was also recapitulated in the B16F10 cells (Figure S10, Supporting Information). The above results indicated that Combo‐NP+L was more potent than PDT‐NP+L in cell killing. After that, the apoptosis of OCM1 and B16F10 cells with various treatments was studied. The results showed that the apoptosis rate of OCM1 treated with Combo‐NP+L (36.2%) was 11.83 times higher than that of OCM1 treated with PBS (3.1%) (Figure 2H,I). Similar results were obtained in B16F10 cells (Figure S11, Supporting Information). Finally, a live and dead cell assay was performed on 3D tumor spheres of B16F10 cancer cells. Results showed that Combo‐NP+L resulted in a higher percentage of dead cells (red) than a single drug did (either Len‐NP or PDT‐NP+L) (Figure S12, Supporting Information). Taken together, the above results demonstrated that Combo‐NP+L was effective in generating ROS, which subsquently triggerd the release of Lenvatinib and killed cancer cells.

### 2.3 Combo‐NP Resulted in a Stronger ICD Effect and more DCs Maturation In Vitro

**Figure 3:**
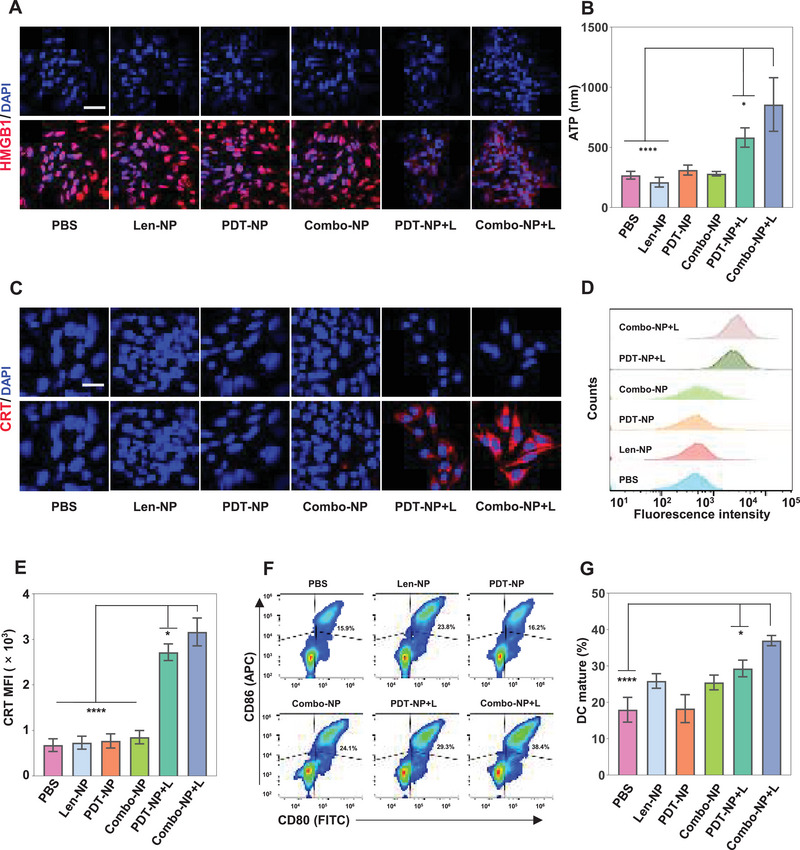
Combo‐NP resulted in a stronger ICD effect and more DCs maturation in vitro. A) Representative CLSM images of release of HMGB1 in OCM1 cells. Scale bar = 50 µm. B) Extracellular ATP levels in OCM1 cells after various treatments. C) Representative CLSM images of the exposure of CRT in OCM1 cells. Scale bar = 20 µm. D) Representative flow cytometric curves and E) the corresponding quantification of surface expression of CRT on OCM1 cells. F,G) Semi‐quantitative study of the maturation of BMDCs co‐cultured with B16F10 cells with various pretreatments by FCM. *n* = 3. Data are presented as mean ± SD. Statistical significances between every two groups were calculated via one‐way ANOVA. * *p* < 0.05, **p < 0.01, ***p < 0.001, ****p < 0.0001.

Recently, an increasing number of studies confirmed that chemotherapeutic agents such as anthraquinones and oxaliplatin could induce ICD in tumor cells.^[^ ^15^ ^]^ In contrast, PDT is able to induce a more intense ICD effect by generating ROS.^[^ ^16^ ^]^ Tumor cells that undergo ICD generate a series of DAMPs, including surface‐exposed calreticulin (CRT), passively released high mobility group box 1(HMGB1), and secreted adenosine triphosphate (ATP), which are the key indicators of ICD.^[^ ^16a^ ^]^ Specifically, CRT is exposed on the cell surface which acts as a “eat‐me signal”, and the ATP serves as “find‐me” signal that jointly triggers phagocytosis of the dying tumor cells by the DCs.^[^ ^17^ ^]^ Moreover, HMGB1 promotes DCs maturation and antigen presentation. These signals together induce immune responses. Therefore, a series of experiments were conducted to study whether Combo‐NP+L could induce ICD effect. First, immunofluorescence staining of HMGB1 (red) in OCM1 and B16F10 was performed. The results showed that the red fluorescence intensity in cells treated with Combo‐NP+L was significantly lower than that of cells treated with others, demonstrating Combo‐NP+L had stronger ability to induce HMGB1 efflux (**Figure** **3**A; Figure S13, Supporting Information). Further, the ATP levels after different treatments were measured. Results indicated that the ATP in the supernatant of OCM1 cells treated with Combo‐NP+L was the highest (856.5 nm), which was almost 3.2 and 1.5 times higher than that of cells treated with PBS (268.4 nm) and PDT‐NP+L (582.4 nm) (Figure 3B). Similar results were also obtained in B16F10 cells (Figure S14, Supporting Information). Third, CLSM results showed that the red fluorescence coming from CRT of OCM1 cells treated with PDT‐NP+L and Combo‐NP+L was significantly higher than that of cells treated with others (Figure 3C). Further, the fluorescence intensity of CRT in cells was quantified by FCM. The results demonstrated that fluorescence intensity of the cells treated with Combo‐NP+L (3163.7) was 4.7 and 1.2 times higher than that of cells treated with PBS (678.7) and PDT‐NP+L (2717.7), respectively (Figure 3D,E). Similar results could be found in B16F10 cells (Figure S15, Supporting Information). Taken together, the above results fully demonstrated that Combo‐NP+L could induce a stronger ICD effect. Finally, to further evaluate whether the ICD effect induced by DAMPs could promote the maturation of bone marrow‐derived dendritic cells (BMDCs), the DCs maturation of different treatments was analyzed by FCM. The results demonstrated that Combo‐NP+L induced more DCs maturation (37%), which was 2.1 and 1.3 times higher than those treated with PBS (18%) and PDT‐NP+L (29.3%), respectively (Figure 3F,G). In conclusion, Combo‐NP+L could result in a stronger ICD effect and more DCs maturation in vitro.

### 2.4 Biodistribution and Anticancer Efficacy of Combo‐NP in OCM1 Tumor‐Bearing Nude Mice

Good biocompatibility and low systemic toxicity are prerequisites for biomedical application. Subsequently, Len‐NP, PDT‐NP, and Combo‐NP were injected through the tail vein of KM mice to evaluate their biosafety. The blood and major organs in each group were collected after 14 days. The results showed there was no significant difference between the body weight of mice treated with Combo‐NP and those treated with PBS (Figure S16, Supporting Information). Next, the physiological and biochemical indexes of the blood of mice were analyzed. The results showed that the blood biochemical parameters were within the normal range in all groups (Figure S17, Supporting Information). Finally, hematoxylin‐eosin (H&E) staining of the major organs of mice treated with different drugs was performed. The results showed there were no significant morphological changes in the organs of mice treated with Combo‐NP compared with those treated with PBS (Figure S18, Supporting Information). The above results fully demonstrated the safety of Combo‐NP.

**Figure 4:**
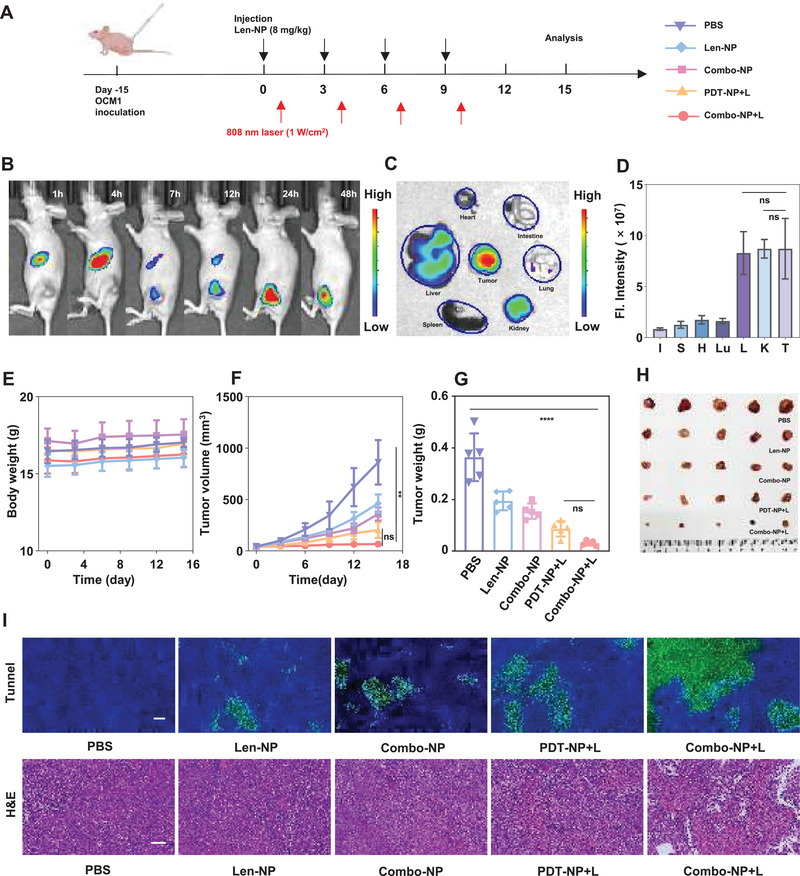
Biodistribution and antitumor efficacy of Combo‐NP on an OCM1 tumor‐bearing nude mice model. A) Schematic illustration of the treatment schedule. B) Biodistribution of Combo‐NP via fluorescence imaging of OCM1 bearing mice after intravenous injection of Combo‐NP@Cy7.5 at different time points. C) *Ex‐vivo* imaging of key organs and tumors. D) The mean fluorescence intensity in major organs and tumor tissues after 48 h intravenous injection. E) Body weight of mice. F) Tumor growth inhibition curve. G) Corresponding tumor weight and H) representative tumor images. I) H&E staining and TUNEL assay, Scale bar = 50 µm. *n* = 5. The data are analyzed by two‐way ANOVA (F) or one‐way ANOVA D,G). * *p* < 0.05, **p < 0.01, ***p < 0.001, ****p < 0.0001.

Subsequently, an OCM1 subcutaneous mice tumor model was constructed to investigate the biodistribution of Combo‐NP in vivo (**Figure** **4**A). The mice were injected with Cy7.5‐labeled Combo‐NP (Combo‐NP@Cy7.5) via tail vein and the fluorescence signal was monitored by In Vivo *Imaging System* (*IVIS Spectrum*). The in vivo imaging showed that the fluorescence intensity of tumor site continued to increase with increasing injection time and reached the peak at ≈24 h. Moreover, a relative strong fluorescence signal at the tumor site still was detected after 48 h (Figure 4B). The above findings demonstrated that Combo‐NP could accumulate effectively at tumor sites. After 48 h of administration, the mice were executed and the major organs and tumors were dissected for *ex vivo* bio‐imaging (Figure 4C), and the fluorescence intensity of tumor was significantly higher than that of heart, lung, spleen, and intestine. The liver and kidney as the main metabolic organs display strong fluorescence intensity in the body (Figure 4D).

Next, BALB/c nude mice with subcutaneous OCM1 tumors were used to evaluate the anti‐tumor activity of Combo‐NP following the treatment schedules (Figure 4A). The results showed that there was no significant difference in body weight of mice between groups till the end of experiments (Figure 4E), further confirming the biosafety of Combo‐NP. In addition, vigorous tumor growth inhibition was observed in mice treated with Combo‐NP+L, with a mean tumor volume of 66.4 mm^3^ at day 15, which was about 1/3 of the mean tumor volume of mice treated with PDT‐NP+L (203.3 mm^3^). On the contrary, the mean tumor volume of mice treated with PBS increased to 862.5 mm^3^ (Figure 4F). Furthermore, the mean tumor weight of mice treated with PBS up to 0.36 g, which was 12 times higher than that of mice treated with Combo‐NP+L (0.03 g) (Figure 4G,H). The above results fully revealed the extraordinary antitumor efficacy of Combo‐NP+L.

Finally, the necrosis and apoptotic death in the tumor tissues were measured by H&E staining and Terminal deoxynucleotidyl transferase‐mediated dUTP biotin nick and labeling (TUNEL). The H&E staining results showed that there was greater nuclear fragmentation, nucleolysis in tumor tissues of mice treated with Combo‐NP+L. Meanwhile, TUNEL assays suggested that the green fluorescence intensity in the tumor tissues of mice treated with Combo‐NP+L was significantly higher than that in the tumor tissues of mice treated with others. Taken together, Combo‐NP+L was the most effective in inhibiting tumor growth (Figure 4I).

### 2.5 Vascular Normalization by Combo‐NP on a B16F10 Subcutaneous Cancer Model

**Figure 5:**
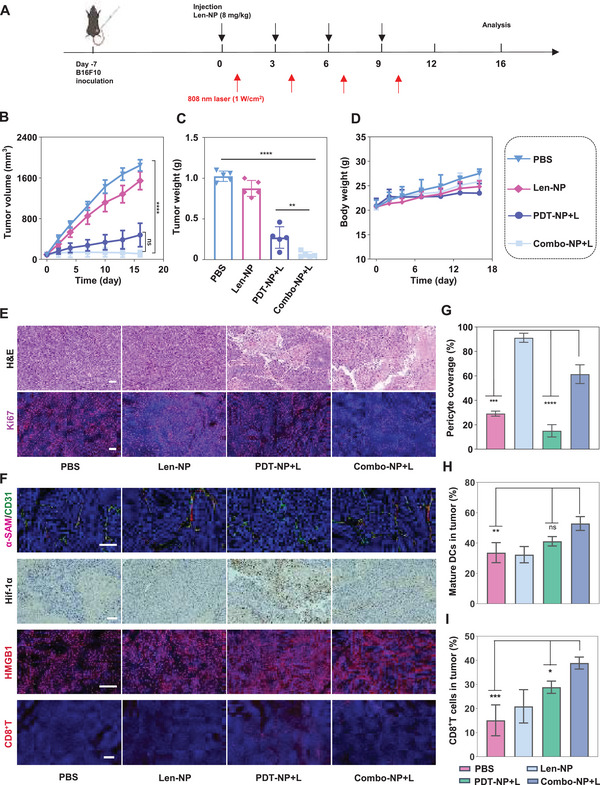
Combo‐NP facilitated the tumor vascular normalization and amplified the photodynamic‐immunotherapy on a B16F10 subcutaneous cancer model. A) Schematic illustration of the treatment schedule. B) Tumor growth inhibition curve and C) tumor weight of mice in different groups. D) Body weight of mice in different groups. E) H&E staining and Ki67 assay in B16F10 tumor tissues. F) Representative immunofluorescence images of Hif‐1α, CD31 (green), α‐SMA (red), HMGB1, and CD8^+^ T cells staining in tumor tissues. Scale bar = 100 µm. G) The proportions of α‐SMA^+^ pericyte‐covered blood vessels. H) The percentage of cell population of matured DCs (CD80^+^CD86^+^) within tumors. I) The percentage of cell population of CD3^+^CD8^+^ T cells within tumors. *n* = 3. The data are analyzed by two‐way ANOVA (B) or one‐way ANOVA (C, D, G, H, I). *p < 0.05, **p < 0.01, ***p < 0.001. ****p < 0.0001.

The antitumor ability of Combo‐NP+L was further examined on B16F10 tumor‐bearing male C57BL/6 mice. The treatment schedule is shown in **Figure** **5**A. The results demonstrated that the tumor volume of mice treated with Combo‐NP+L was almost completely reduced on 16th day. Specifically, the average tumor volume of mice treated with Combo‐NP+L was only 111 mm^3^. While the tumor volume of mice treated with PDT‐NP+L and PBS reached up to 480 mm^3^, 1827 mm^3^, respectively (Figure 5B). Finally, the mice were sacrificed and the tumors were isolated and weighted. The results demonstrated that tumor weight of mice treated with Combo‐NP+L (1.02 g) was almost 1/17 of tumor weight of mice treated with PBS (0.06 g) (Figure 5C). Taken together, the above results revealed that Combo‐NP+L had the strongest ability to inhibit the tumor growth, and there was no decreasing trend in body weight of mice (Figure 5D). Further, the necrosis and cell proliferation in the tumor tissues were measured by H&E and Ki‐67 (red fluorescence). The results showed that there was the most severe damage and the weakest cell proliferation in the tumor tissues of mice treated with Combo‐NP+L (Figure 5E). The above results indicated that Combo‐NP+L had the strongest ability to suppress the tumor growth.

Subsequently, the tumor vascular normalization was studied. It is well known that abnormal tumor vascular aggravates hypoxia tumor microenvironment, which strongly diminishes the PDT effect.^[^ ^7^, ^18^ ^]^ Moreover, abnormal vasculature decreases the infiltration of CTLs and increases the immunosuppressive microenvironment.^[^ ^19^ ^]^ However, Lenvatinib, an anti‐angiogenesis inhibitor, can normalize blood vessels.^[^ ^10^ ^]^ Normalized vasculature with high pericyte and low micro‐vessel density can alleviate immunosuppression and improve the efficacy of PDT. Therefore, it was hypothesized that Combo‐NP+L could facilitate the vascular normalization and therefore amplify photodynamic‐immunotherapy. To explore this, first, the pericyte and micro‐vessel density were detected by α‐Smooth Muscle Actin (α‐SMA) (red) and Platelet endothelial cell adhesion molecule‐1 (CD31) (green) immunostaining assay, respectively. The results showed that the pericyte coverage of tumor vessels (a marker of tumor vascular normalization) in mice treated with Len‐NP and Combo‐NP+L, which reflect tumor vascular perfusion function, was significantly increased from 15.1% to 91.2% and 61.3% compared to PDT‐NP+L, respectively. (Figure 5F,G). Subsequently, hypoxia signal in tumor tissues was used to further assess the ability of vasculature normalization. The hypoxia inducible factor‐1α (Hif‐1α) was used to detect the hypoxia region, and the results showed the hypoxia signal in tumor tissues of mice treated with Len‐NP and Combo‐NP+L was dramatically reduced compared with that in tumor tissues of mice treated with PDT‐NP+L (Figure 5F). The above results fully demonstrated that Combo‐NP could normalize the tumor vasculature. In addition, immunofluorescence of HMGB1 (red) and CD8^+^ T (red) showed that the Combo‐NP+L induced the strongest release of HMGB1 from the nucleus, which was corresponding to the results of in vitro experiments. The red fluorescence intensity (CD8^+^ T cells) was significantly higher in the tumor tissues of mice treated with Combo‐NP+L than those in the other treatments (Figure 5F). In summary, the above results revealed that Combo‐NP+L could alleviate the hypoxic state of tumor microenvironment and increase the infiltration of CD8^+^ T cells by remodeling and normalizing tumor vasculature, thereby amplifying the photodynamic‐immunotherapy.

As previously described, Combo‐NP+L generated ROS to induce ICD, which subsequently enhanced the maturation of DCs and recruitment of CTLs in the tumor microenvironment, thereby turning “cold tumor” into “hot tumor” with better antitumor immune responses. In other words, Combo‐NP+L could reprogram the immunosuppressive microenvironment by normalizing the vascular, thereby increasing the infiltration of CTLs and reducing the proportion of negative‐regulatory immune cells, such as MDSC (myeloid‐derived suppressor cells), Tregs (regulatory T cells) and M2 macrophages. Therefore, the maturation of DCs in mice tumors and Lymph nodes were first analyzed by FCM. The results showed that there was higher proportion of matured DCs in tumors and lymph nodes of mice treated with Combo‐NP+L than those treated with PBS, increasing from 33.7% to 52.9% in the tumors and 14.5% to 32.7% in the lymph nodes, respectively (Figure 5H; Figure S19, Supporting Information), indicating that there was the strongest ability of Combo‐NP+L to activate the T cells and enhance the CD8^+^ T cells infiltration. As shown in Figure 5I, the proportion of CD8^+^ T cells in the tumor of mice treated with Combo‐NP+L was the highest (38.9%), which was 1.4‐fold higher than that in the tumor of mice treated with PDT‐NP+L (28.8%). Meanwhile, Combo‐NP+L (36.6%) resulted in 1.9‐fold and 1.4‐fold higher CD8^+^ T cells infiltration in the spleen than PBS (19.5%) and PDT‐NP+L (26.9%), respectively (Figure S20, Supporting Information), indicating there was an activated immune response in TME. Finally, Tregs, M2 macrophages and MDSC at the tumor tissues were analyzed. The results showed that Tregs, MDSC, and M2 in the tumor of mice treated with Combo‐NP+L as compared to PBS decreased from 54.2% to 18.8%, from 28.3% to 14.2% and from 61.3% to 17.8%, respectively (Figure S21, Supporting Information). Taken together, Combo‐NP not only induced ICD effects via ROS generation, but also regulated the immunosuppressive tumor microenvironment by encapsulating Lenvatinib, thereby amplifying the photodynamic‐immunotherapy effect.

### 2.6 Combo‐NP Upregulates the Expression of PD‐L1 in Melanoma

**Figure 6:**
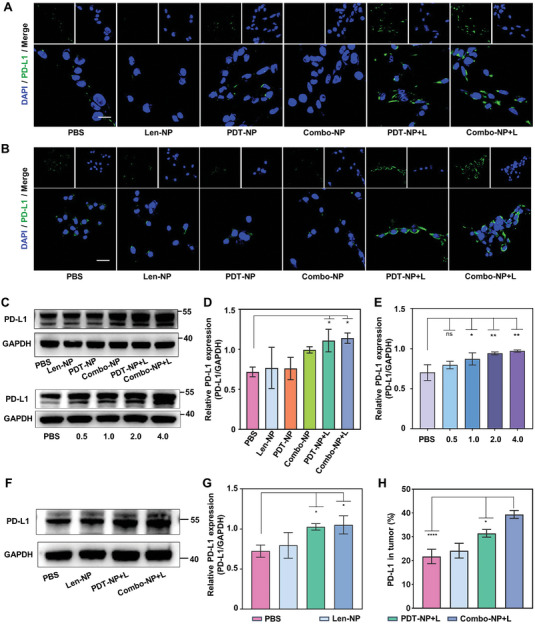
Combo‐NP upregulates the expression of PD‐L1 in melanoma. A) Representative CLSM images of PD‐L1 in OCM1 cells. Scale bar = 20 µm. B) Representative CLSM images of PD‐L1 in B16F10 cells. Scale bar = 20 µm. C) Representative western blot of PD‐L1 in OCM1 cells treated with different formulations and different light time. D) Semi‐quantification of PD‐L1 with the GAPDH loading control in cells treated with Len‐NP, PDT‐NP, Combo‐NP, PDT‐NP+L, and Combo‐NP+L. E) Semi‐quantification of PD‐L1 in cells treated with Combo‐NP+L after different light time. F) Representative western blot of PD‐L1 in B16F10 tumors treated with different formulations. G) Semi‐quantification of PD‐L1 with the GAPDH loading control in tumors treated with Len‐NP, PDT‐NP+L, and Combo‐NP+L. H) The percentage of positive of PD‐L1 within tumors. *n* = 3. Data are presented as mean ± SD. Statistical significances between every two groups were calculated via one‐way ANOVA. * *p* < 0.05, **p < 0.01, ***p < 0.001, ****p < 0.0001.

In recent years, immune checkpoint blockade (ICB) has emerged as a prospective cancer therapy strategy. The programmed cell death factor‐1 (PD‐1) /PD‐L1 immune checkpoint pathway inhibits the proliferation and activation of T cells, thereby fostering tumor immune escape.^[^ ^20^ ^]^ Recent studies have demonstrated that anti‐PD‐L1 monoclonal antibody can block the interaction of PD‐1 and PD‐L1 to restore the function of T cells in the TME. However, anti‐PD‐L1 monoclonal antibodies are effective in only 15–20% of patients because most cancer is considered as “cold tumor” with limited infiltration of anti‐tumor immune cells and a low rate of positive PD‐L1 expression.^[^ ^1^, ^21^ ^]^ The above experiments unveiled that Combo‐NP+L could recruit CTLs to tumor sites, while alleviating the immunosuppressive microenvironment. Moreover, some studies showed that PDT can stimulate the expression of PD‐L1.^[^ ^22^ ^]^ We examined whether Combo‐NP+L could synergize with anti‐PD‐L1 antibody to increase the efficacy of immunotherapy. First, the expression of PD‐L1 in OCM1 and B16F10 was assessed in vitro. The CLSM results showed that there was a significant increase in green fluorescence intensity on OCM1 and B16f10 cells treated with PDT‐NP+L and Combo‐NP+L compared to those treated with PBS (**Figure** **6**A,B). Next, western blot analysis (Figure 6C,D) demonstrated a substantial 39% and 42% elevation in PD‐L1 expression in cells treated with PDT‐NP+L and Combo‐NP+L, respectively, relative to PBS‐treated cells. Notably, Len‐NP and PDT‐NP treatment showed no evident effects on the expression of PD‐L1. Subsequently, the expression of PD‐L1 in cells treated with Combo‐NP+L after different light time was examined. The results revealed a time‐dependent increase in PD‐L1 expression, with cells irradiated for 1 and 2 minutes showing a 17% and 24% augmentation, respectively, compared with cells without irradiation (Figure 6E). The above results indicate that the Combo‐NP can induce the expression of PD‐L1 in a time‐dependent manner.

Further investigations extended to evaluating PD‐L1 expression within tumor tissues. Western blot analyses (Figure 6F,G) demonstrated a significant 30% and 32% enhancement in PD‐L1 expression in tumors treated with PDT‐NP+L and Combo‐NP+L, respectively, in comparison to tumors treated with PBS. The expression of PD‐L1 in tumor tissues was additionally assessed by FCM, and the results showed that the expression of PD‐L1 in B16F10 tumor tissues of mice treated with Combo‐NP+L was 2 times higher than that of mice treated with PBS (Figure 6H; Figure S22, Supporting Information). Collectively, these experiments demonstrate that Combo‐NP+L not only improve the immunosuppressive microenvironment, but also increase the expression of PD‐L1. Therefore, Combo‐NP holds promise as a potential immune adjuvant to enhance anti‐PD‐L1 efficiency.

### 2.7 Combo‐NP Combined with α‐PD‐L1 mAb Induced Systemic Antitumor‐Mediated Abscopal Effect and Inhibited Metastasis

**Figure 7:**
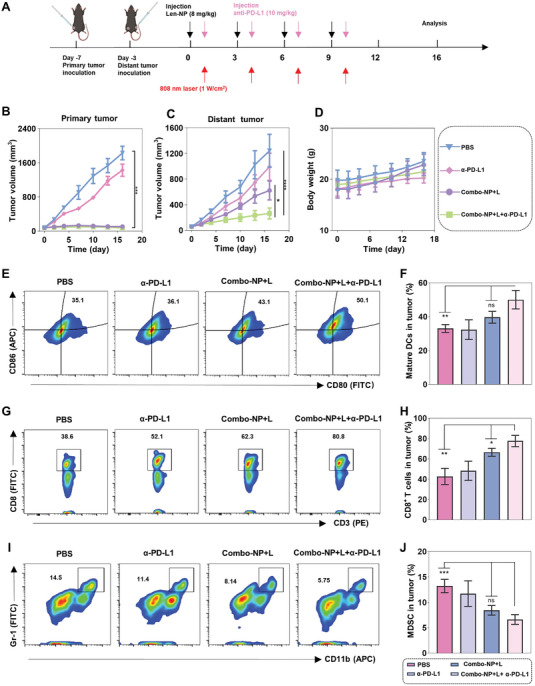
The combination of Combo‐NP and α‐PD‐L1 mAb induced systemic antitumor‐mediated abscopal effect on a B16F10 subcutaneous cancer model. A) Treatment schedule of an abscopal cancer mice model after various treatments. B) Tumor growth curve of primary and C) distant tumors. D) Tumor weight of different groups after 16 days treatment. E) Representative flow cytometric plots of matured DCs (CD80^+^ CD86^+^) and F) percentages of matured DCs in the distant tumors. G) Representative flow cytometric plots of CD3^+^CD8^+^T cells and H) percentages of CD3^+^CD8^+^T cells in the distant tumors. I) Representative flow cytometric plots of MDSC (CD11b^+^ Gr‐1^+^) and J) percentages of MDSC (CD11b^+^ Gr‐1^+^) in the distant tumors. *n* = 3. The data are analyzed by two‐way ANOVA (B,C) or one‐way ANOVA (F,H,J) *p < 0.05, **p < 0.01, ***p < 0.001, ****p < 0.001.

Subsequently, whether the combination of Combo‐NP and α‐PD‐L1 mAb could induce a systemic antitumor‐mediated abscopal effect and inhibit tumor metastasis was further studied. To verify this hypothesis, first, a bilateral B16F10 syngeneic model was established to study the tumor growth and immune profiles of abscopal tumor. As shown in **Figure** **7**A, the primary tumor was constructed subcutaneously on the left flank of mice. Four days later, the second tumor was inoculated on the right flank to simulate a metastatic tumor.^[^ ^23^ ^]^ When the volume of the primary tumor reached ≈80 mm^3^, the mice were randomly divided into four groups (n = 5 per group) and treated with PBS, α‐PD‐L1, Combo‐NP+L, and Combo‐NP+L+α‐PD‐L1, respectively. 808 nm laser irradiation was required for laser treatment of primary tumors in mice. The results demonstrated that the bilateral tumors in the mice treated with PBS grew rapidly. Moreover, it seemed that α‐PD‐L1 alone slightly inhibited the tumor growth on both sides. However, the primary tumors of mice treated with Combo‐NP+L and Combo‐NP+L+α‐PD‐L1 were almost completely inhibited. Notably, for distant metastatic tumors, the metastatic tumor volume of mice treated with Combo‐NP+L (623.5 mm^3^) was ≈2.4 times larger than that of mice treated with Combo‐NP+L+α‐PD‐L1 (265 mm^3^). The above results suggested that the triple combination therapy of Combo‐NP+L+α‐PD‐L1 could inhibit the growth of primary and metastatic tumors (Figure 7B,C). Moreover, there was no significant difference in the body weight of mice in each treatment group (Figure 7D). To further investigate the underlying mechanism of the synergistic anti‐tumor effect of Combo‐NP+L+α‐PD‐L1, the mice were executed on day 16 and immune cells in secondary (distant metastatic) tumors were analyzed by FCM. The results demonstrated that the tumors of mice treated with Combo‐NP+L+α‐PD‐L1 had the highest maturation rate of DCs (50%), which was 1.3 times higher than that of mice treated with Combo‐NP+L (39.7%) (Figure 7E,F). Furthermore, the CD8^+^ T cells in tumors of mice treated with Combo‐NP+L+α‐PD‐L1 had up to 77.6%, which was 1.2 times higher than that of mice treated with Combo‐NP+L (66.5%) (Figure 7G,H). Finally, the percentage of immunosuppressive including MDSCs and Tregs was further explored, and the results showed that MDSCs and Tregs in the mice treated with Combo‐NP+L+α‐PD‐L1 as compared to PBS decreased from 13.2% to 6.6% and from 15.6% to 7.6%, respectively (Figure 7I,J; Figure S23, Supporting Information). Subsequently, a delayed lung metastasis cancer model was established by intravenously injecting cancer cells 4 days after primary tumor induction to evaluate the ability of the triple‐combination therapy (Combo‐NP+L+α‐PD‐L1) to inhibit the lung metastasis. After 20 days treatment schedule (Figure S24A, Supporting Information), the mice were executed and lungs were obtained. The results demonstrated that although there were visible metastatic nodules in the lungs of mice with other treatments, there were no metastasis nodules in the lungs of mice treated with the Combo‐NP+L+α‐PD‐L1 (Figure S24B, Supporting Information). In summary, Combo‐NP+L could fully activate the anti‐tumor immune responses for photodynamic‐immunotherapy and improve the immunosuppressive tumor microenvironment through vascular normalization and immunomodulation. Further combination of anti‐PD‐L1 mAb activated greater systemic immune activity to exhibit stronger abscopal effects and inhibit tumor metastasis.

## 3 Conclusions

PDT has been proposed as a treatment modality for UM due to its proven effectiveness and non‐invasiveness nature. However, the presence of an abnormal vascular system limits the PDT effect by exacerbating the tumors hypoxic microenvironment. Moreover, abnormal vasculature was a major metastasis pathway for uveal melanoma. Fatal tumor metastasis was directly related to the survival of UM patients. Recently, adoptive transfer of tumor‐infiltrating lymphocytes and DC vaccination loaded with tumor‐specific peptides have been applied to metastatic melanoma.^[^ ^24^ ^]^ However, there is still no systemic treatment modality that targets both primary and metastatic melanoma.

This study introduces a NIR‐II theranostic Combo‐NP for photodynamic‐immunotherapy via normalizing blood vessels was designed. PDT is effecive in directly eliminating tumors directly through the generation of ROS and the induction of ICD which initiated immune reactions. Moreover, Combo‐NP could alleviate hypoxia by normalizing the tumor vessels, thus improving the efficiency of PDT, most importantly, normalized vascular could increase the infiltration of CTLs at the tumors and reprogram the immunosuppressive microenvironment. These effects combined to amplify the photodynamic‐immunotherapy. The in vitro studies showed that the anticancer effect of Combo‐NP+L on OCM1 (IC_50_ = 2.3 µg mL^−1^) and B16F10 (IC_50_ = 3.4 µg mL^−1^) cells was remarkably improved. In vivo on OCM1 and B16F10 animal model, the tumor inhibition rates of mice treated with Combo‐NP+L were 91% and 94%, respectively. Moreover, there was lower hypoxia signal and the more infiltration of CTLs in the tumors of mice treated with Combo‐NP+L. In combination with anti‐PD‐L1 mAbs, the antitumor effects produced by local treatments could be extended to whole body, inducing a systemic anti‐tumor immune response that mediated distant effects and inhibited tumor metastasis. In conclusion, Combo‐NP+L represents a novel and promising strategy for treating UM. It also serve as a potential immune adjuvant to enhance the efficiency of anti‐PD‐L1 therapy, thereby offering a new therapeutic paradigm in the battle against UM and other related cancers.

## Conflict of Interest

The authors declare no conflict of interest.

## Supporting information

Supporting Information

## Data Availability

The data that support the findings of this study are available from the corresponding author upon reasonable request.
