# Supplementary material for: Near‐Infrared‐II Nanoparticles for Vascular Normalization Combined with Immune Checkpoint Blockade via Photodynamic Immunotherapy Inhibit Uveal Melanoma Growth and Metastasis
Source: Adv Sci (Weinh). 2023 Nov 8;10(35):2206932. doi: 10.1002/advs.202206932 (PMC10724444; doi:10.1002/advs.202206932)
Supplement: Supplementary file 1 — Supporting Information [file ADVS-10-2206932-s001.pdf]

ADVANCED SCIENCE
Open Access

Supporting Information

for *Adv. Sci.*, DOI 10.1002/advs.202206932

Near-Infrared-II Nanoparticles for Vascular Normalization Combined with Immune Checkpoint Blockade via Photodynamic Immunotherapy Inhibit Uveal Melanoma Growth and Metastasis

Xiaoqin Zheng, Yunyi Shi, Dongsheng Tang, Haihua Xiao, Kun Shang, Xuezhi Zhou* and Gang Tan**

Supporting Information

Near-Infrared-II Nanoparticles for Vascular Normalization Combined with Immune Checkpoint Blockade *via* Photodynamic Immunotherapy Inhibit Uveal Melanoma Growth and Metastasis

Xiaoqin Zheng [1], *Yunyi Shi* [1], *Dongsheng Tang* [2,3], *Haihua Xiao* [2,3], *Kun Shang* [4,*], *Xuezhi Zhou* [5,*], *Gang Tan* [1,*]

1. Department of Ophthalmology, The First Affiliated Hospital, Hengyang Medical School, University of South China, Hengyang, 421001 Hunan China.
2. Beijing National Laboratory for Molecular Sciences, State Key Laboratory of Polymer Physics and Chemistry, Institute of Chemistry, Chinese Academy of Sciences, Beijing, 100190, P.R. China.
3. University of Chinese Academy of Sciences Beijing 100049, P. R. China.
4. Institute of Medical Technology, Peking University Health Science Center, Beijing, 100190, P. R. China.
5. Eye Center of Xiangya Hospital, Central South University, Changsha, Hunan, 410008, P.R. China

TABLE OF CONTENTS

Materials and Methods....4
Figure S1....13
Figure S2....14
Figure S3....15
Figure S4....16
Figure S5....17
Figure S6....18
Figure S7....19
Figure S8....20
Figure S9....21
Figure S10....22
Figure S11....23
Figure S12....24
Figure S13....25
Figure S14....26
Figure S15....27
Figure S16....28
Figure S17....29
Figure S18....30
Figure S19....31
Figure S20....32
Figure S21....33
Figure S22....34
Figure S23....35
Figure S24....36
Table S1....37

References..38

1. Supplemental materials and methods

1.1 Materials and reagents

The ROS sensitive polymer (P1) was synthesized as previously described.[1] Trifluoroacetic acid, trichloro(phenyl)silane, boron (III) fluoride ethylether complex, 4-bromobenzaldehyde, beta-alanine, 5-bromo-2-thiophenecarboxylic acid, bis(2-hydroxyethyl) disulfide and DSPE-PEG_{2000} were purchased from Energy Chemical, shanghai, China. 4-[3-chloro-4-(cyclopropylcarbamoylamino)phenoxy]-7-methoxyquinoline-6-carboxamide (defined as Lenvatinib), 2,5-Bis(2-ethylhexyl)-3,6-bis(5-(trimethylstannyl)thiophen-2-yl)pyrrolo[3,4-c]pyrrole-1,4(2H,5H)-dione (defined as compound 1) and 4,4''-Dibromotriphenylamine (defined as compound 2) were purchased from Alfa (Zhengzhou, China). 1,3-diphenylisobenzofurane (DPBF) was purchased from Aladdin (Shanghai, China). All chemicals and regents were obtained commercially and used without further purification unless otherwise noted.

3-(4,5-dimethylthiazol-2-yl)-2,5-diphenyltetrazolium bromide (MTT) was purchased from Aladdin (Shanghai, China). Dulbecco's modified Eagle's medium (DMEM) with 4.5 g glucose, penicillin/streptomycin (P/S), trypsin-EDTA (0.25%) and Fetal Bovine Serum (FBS) were purchased from Gibco (Gran Island, NY, USA). 2-(4-amidinophenyl)-1H-indole-6-carboxamidine (DAPI) and TUNEL Apoptosis Assay Kit were purchased from Solarbio Science & Technology Co., Ltd. (Beijing, China). Annexin V-FITC apoptosis detection kit was purchased from Elabscience.

1.2 General measurements

^{1}H NMR spectra was measured by a 300 MHz and 400 MHz NMR spectrometer (Bruker, Germany) at room temperature. The morphology and size of nanoparticles were measured by transmission electron microscope (TEM) carried out with a HT7700 electron microscope. Size and zeta potential measurements were conducted *via* a Malvern Zetasizer (Nano ZS, UK). The absorption spectrum was measured using an ultraviolet-visible spectrometer (UV-vis, UV-2600, Shimadzu, Japan). Immunofluorescence images were conducted using a confocal laser scanning microscope (CLSM, FV1000-IX8l, Olympus, Japan). The MTT assay was conducted using a Microplate reader (SpectraMax M3). The mice imaging was conducted by an *In Vivo Imaging System* (*IVIS*, PerkinElmer). Flow cytometry (FCM) was done by a flow cytometry analyzer (Beckman Coulter, USA).

1.3. Methods

1.3.1. Synthesis of Monomer 2

The Monomer 2 was synthesized as previously described.[2] To a solution of CF_3COOH (5.75 g, 50.44 mmol), 2,4-dimethylpyrrole (10.0 g, 104.46 mmol) in dry DCM (30 mL) was slowly added $PhSiCl_3$ (10.67 g, 36.19 mmol). The mixture was stirred at room temperature for 10 min. Next, the NEt_3 (15 mL) was added and stirred at room temperature for another 15 min. Then, the boron (III) fluoride ethylether complex (75 mL) was added and stirred at room temperature for 3 h. When TLC analysis indicated that the reaction was complete, the mixture was diluted with DCM (100 mL × 3), washed with water (200 mL) and saturated NaCl solution (100 mL). The organic layer was dried over anhydrous $MgSO_4$, filtered, and concentrated *in vacuo*. The crude product was purified by silica gel column chromatography to give monomer 2 as a reddish brown solid (3.41g, 21.4% yield). ^{1}H NMR (400 MHz, $CDCl_3$): δ ppm 6.14 (s, 2H), 2.53 (s, 6H), 2.29 (s, 6H). ^{13}C NMR (100 MHz, $CDCl_3$): δ ppm 171.06, 162.87, 159.64, 62.03, 36.78, 31.74, 26.62, 25.27.

1.3.2. Synthesis of compound 3

The compound 3 was synthesized as previously described.[2] To a solution of monomer 2 (100.0 g, 0.15 mmol) and 4-bromobenzaldehyde (83.3 mg, 0.45 mmol) in toluene (10 mL) was added beta-alanine (10 mg) and piperidine (20 μL). The mixture was stirred at 80 °C for 24 h. Then, the solvent was evaporated to afford the crude product, which was purified by silica gel column chromatography to give compound 3 as a dark green solid (84.0 mg, 86.4% yield). ^{1}H NMR (400 MHz, DMSO-d_6): δ ppm 7.78 (d, 2H), 7.69 (d, 4H), 7.61 (d, 4H), 7.56 (d, 2H), 7.30 (s, 2H), 2.35 (s, 6H). ^{13}C NMR (100 MHz, DMSO-d_6): δ ppm 154.90, 154.68, 152.01, 144.09, 141.75, 139.41, 135.52, 132.73, 129.99, 123.86, 122.70, 118.68, 16.02.

1.3.3. Synthesis of TPA-BD

Compound 4 was synthesized with the guidance of the previous report.[3] Compound 1 (385.1 mg, 0.45 mmol), compound 2 (72.6 mg, 0.18 mmol), compound 3 (117.0 mg, 0.18 mmol), compound 4 (47.9 mg, 0.09 mmol). P(*o*-tol)$_3$ (11.0 mg, 0.036 mmol), and dba_3Pd_2 (8.2 mg, 0.009 mmol) were added into a 25 mL Schlenk tube equipped with a magnetic stirrer. Toluene (15 mL) was then added into the tube, and the solution was deoxidized with nitrogen for 5 min. The polymerization reaction was carried out at 120 °C under nitrogen atmosphere in dark for

24 h. After cooling down, the crude product was filtered by AlO_3 to remove the solid catalyst, and then purified by sedimentation in methanol. The black solid of TPA-BD was obtained.

1.3.4. Formulation of Len-NP, PDT-NP and Combo-NP

Briefly, the TPA-BD polymer (10 mg) was dissolved in THF (5 mL), P1(50 mg) and mDSPE-PEG_{2000} (50 mg) were completely dissolved in DMSO (3 mL), and then added into 40 mL pure water under continuous sonication. Lenvatinib (10 mg), P1(50 mg) and DSPE-PEG_{2000} (50 mg) were dissolved in DMSO (4 mL), and then added dropwise into 20 mL pure water with magnetic stirring. Afterward, the organic solvent was cleared by dialysis for 24 hours and then PDT-NP, Len-NP were obtained. The Combo-NP solution was a mixture of PDT-NP and Len-NP in certain ratio. All nanoparticle solutions were stored at 4 °C for further use.

1.3.5. Singlet oxygen generation of PDT-NP

The singlet oxygen ($^1O_2$) generation ability by PDT-NP under the 808 nm irradiation was detected using the chemical probe 1,3- diphenylisobenzofuran (DPBF), which reacted irreversibly with $^1O_2$ to cause a decrease in the DPBF absorption at about 410 nm. Briefly, the absorbance of DPBF at 410 was adjusted to about 1.0, the absorbance of PDT-NP was adjusted to about 0.2. Then the cuvette was irradiated (808 nm, 1 W/cm^2). Characteristic absorbance at 410 nm was recorded by Microplate reader (SpectraMax, USA).

1.3.6. Cell culture

OCM1 and B16F10 cells were cultured in DMEM (glucose 4.5 g/L) supplemented with 10% fetal bovine serum and 1% P/S at 37 °C with 5% CO_2. When cell fusion reached 80%-90%, the cells were digested with 0.25% trypsin, and then passaged and cultured or inoculated into cell plates for subsequent experiments.

1.3.7. Cellular internalization analysis of nanoparticles

Cover slides were placed in the bottom of each well of a 24-well plate. OCM1 cells (1×10^5) in 1 mL media were added to each well and incubated at 37 °C for overnight. Then the cells were treated with Combo-NP at the concentration of PDT-NP 5 μg/mL for 1 h, 3 h or 6 h respectively. After being washed with cold PBS for three times, the cell was fixed with paraformaldehyde and cell nuclei was stained with DAPI. Subsequently, images were collected by CLSM (DAPI, λ_{ex} = 405 nm, Combo-NP, λ_{ex} = 808 nm). The CLSM imaging system was

FLIM-confocal-AFM (Multi-dimensional confocal microscopy fluorescence imaging system, light guide, up-conversion), produced by the U.S. ISS company. Furthermore, the internalization was further detected and quantified by FCM. OCM1 cells (20 × 10^5) in 1 mL media were added to each well and incubated at 37 °C for overnight. Then the cells were treated with Combo-NP@Cy5.5 for 1 h, 3 h or 6 h respectively. Then washed with PBS and analyzed by FCM.

1.3.8. Cellular ROS generation

Intracellular ROS level was investigated by using DCFH-DA as a fluorescent probe. In brief, cover slides were placed into the bottom of each well of a 24-well plate. OCM1 cells (1 × 10^5) in 1 mL media were added to each well and incubated at 37 °C overnight and then treated with Len-NP, PDT-NP and Combo-NP at the same concentration of photosensitizer (10 μg/mL). After 6 h treatment, cells were washed and then cultured in serum-free medium with DCFH-DA (10 μM) for 20 mins, then the cells of laser groups were irradiated with 808 nm laser. Afterward, the cells were fixed with paraformaldehyde and cell nuclei were stained with DAPI. Subsequently, images were collected with CLSM. Furthermore, the intracellular ROS level was further detected and quantified by FCM. Firstly, cells were seeded in 12-well plate at a density of 2 × 10^5 per well and incubated at 37 °C overnight. Afterward, the cells were treated with the same approach and conditions as the above CLSM analysis. Finally, the cells were harvested immediately to examine the intracellular ROS by FCM.

1.3.9. Cytotoxicity assay *in vitro*

OCM1 and B16F10 cells were seeded in 96-well plates (6 × 10^3 cells per well) and cultured with complete medium (10% FBS) at 37 ℃ for overnight. Then the cells were divided into 6 groups including PBS, Len-NP, PDT-NP, Combo-NP, PDT-NP+L and Combo-NP+L (Lenvatinib and photosensitizer concentrations ranging from 1.3 μM to 20 μM and 0.6 μg/mL to 10 μg/mL, respectively) and incubated for another 12 h. The cells in the laser groups were irradiated with NIR light of 808 nm at power of 1.0 W/cm^2 for 2 min. Finally, the cell viability was determined using MTT assay. For live/dead cell staining of 3D tumor spheroids, 1% agarose gel solution (50 μL) was added to each well. Next, 1500 cells (200 μL complete medium) were added into each well. The cell spheres were basically formed after 7 days, then different formulations were added and incubated another 12 h. The cells of laser groups were irradiated with a NIR light of 808 nm at intensity of 1.0 W/cm^2 for 2 min. Cells were continued to be cultured for 12 h and then washed with PBS. Afterwards, cells were successfully stained

with PI base on the manufacturer's instruction of the live/dead cell staining kit, respectively. Lastly, the cells were imaged by CLSM with Z-stack scanning.

1.3.10. Apoptosis analysis *in vitro*

Cellular apoptosis was assessed with an Annexin V-FITC apoptosis detection kit according to the manufacture's instruction. OCM1 and B16F10 cells were cultured in 12-well plates (2 × 10^5 cells per well) overnight. Afterwards, the cells were treated with PBS, Len-NP, PDT-NP, Combo-NP, PDT-NP+L and Combo-NP+L (Lenvatinib and photosensitizer concentration of 5 μM and 2.5 μg/mL) for 12 h. The cells in the laser groups were irradiated with NIR light of 808 nm and cultured for another 12 h. Finally, the cells were incubated with Annexin/PI reagent in the dark for 20 min and immediately measured with FCM.

1.3.11. ICD induced by Combo-NP *in vitro*

To determine different formulations-induced immunogenic cell death (ICD) of the tumor cells, the secretion of adenosine triphosphate (ATP), calreticulin (CRT) exposure, and extracellular release of high mobility group box 1 (HMGB1) were examined *in vitro*. In order to study the exposed CRT on the cell surface, cover slides were placed into the bottom of each well of a 24-well plate. The cells (1 × 10^5) in 1 mL media were added to each well and incubated at 37 °C overnight , and then the cells were treated with PBS, Len-NP, PDT-NP, Combo-NP, PDT-NP+L and Combo-NP+L for 6 h (Lenvatinib and photosensitizer concentration of 5 μM and 2.5 μg/mL). Further incubated with primary CRT antibody for overnight at 4 °C, and then incubated with the 555-conjugated secondary antibody. Finally, the cells were stained with DAPI, and observed by CLSM. For FCM analysis, cells were seeded in the 12-wells plate at a density of 20 × 10^5 cells/well overnight. Then the cell treatment was the same as CLSM analysis. Next, the cells were washed with PBS and incubated with CRT antibody. After that, the cells were incubated with secondary antibody for 30 min and then analyzed by FCM.

For evaluating intracellular HMGB1, cover slides were placed into each well of 24-wells plate and cells (1 × 10^5) in 1 mL complete media were added. After 12 h incubation, the cells were treated with different formulations like the CRT analysis for 24 h. Next, the cells were washed with PBS and fixed with 4% paraformaldehyde for 15 min, permeabilized with 0.1% Triton X-100 for 10 min, incubated with 1% fetal bovine serum for 30 min. After that, the cells were incubated with primary HMGB1 antibody overnight at 4 °C, and then incubated with secondary antibody. Finally, the cells were examined by CLSM.

Extracellular secretion of ATP was measured with ATP assay kit according to the manufacture's instruction. The cells were seeded in 12-well plate at a density of 20 × 10^5 cells per well and incubated overnight. Subsequently, the cells were treated with different formulations like the CRT analysis for 24 h. Then the culture medium was collected, 100 μL of ATP detection regents was added into wells and incubated 5 min. Then 20 μL of samples were added to each well and blended quickly. The luminescence of the samples was measured by a microplate reader (SpectraMax M3).

1.3.12. BMDC mature *in vitro*

To evaluate dendritic cells (DCs) activation *in vitro*, bone-marrow derived dendritic cells (BMDCs) were obtained from C57BL/6 mice. B16F10 cells were pretreated with PBS, Len-NP, PDT-NP, Combo-NP, PDT-NP+L and Combo-NP+L (Lenvatinib concentration of 5 μM) and then co-incubated with BMDCs for 24 h. After that, DCs were stained with anti-CD11c-PE, anti-CD80-FITC, and anti-CD86-APC for DCs mature analysis by FCM.

1.3.13 Western bloting

All the samples were washed with clod PBS and lysed with RIPA buffer (Biotech) supplement with PMSF (Biotech) and Phosphatase inhibitor cocktail (Biotech) on ice to extract protein. The bicinchoninic acid protein assay kit (Beyotime) was used to measure the concentration of protein. Proteins in equivalent amounts were then separated on a 10% sodium dodecyl sulfate-polyacrylamide gel electrophoresis (SDS-PAGE) and transferred to the PVDF membrane, followed by blocking in TBS-T solution containing 5% skim milk (for phosphorylated proteins) for 1 h. Subsequently, the membrane was incubated with primary antibody against PD-L1 (AF7710, Beyotime) and GAPDH (AF1186, Beyotime) overnight at 4 ℃. Then, second antibodies were added for 1 h at room temperature, and the protein blots were visualized via X-ray film.

1.3.14. Animal welfare and protocols

Healthy KM mice (6-8 weeks old), male C57BL/6 mice, and BALB/c nude mice were purchased from SPF Biotechnology (Beijing, China) and raised in SPF animal rooms. All animal experiments reported herein were performed under guidelines evaluated and approved by Peking University Institutional Animal Care and Use Committee (LA2021316)

1.3.15. *In vivo* biosafety evaluation

Healthy KM mice were randomly divided into 4 groups (n = 3 mice per group). On day 0, 3, 6, and 9, each group mice were injected with PBS, Len-NP, PDT-NP and Combo-NP (photosensitizer concentration of 10 mg/kg, Lenvatinib concentration of 8 mg/kg) through tail vein, respectively. Then the mice were sacrificed at 14 days after treatments, and the blood samples of the mice were collected for hematological and serum biochemical analysis. Meanwhile, the main organs including heart, liver, spleen, lung and kidney were also obtained for further analysis. All the tissues were paraffin embedded and tissue sections were prepared. Then H&E staining were performed to observe pathological features, and images were captured using an EVOS XL Core optical microscope (EVOS XL Core, AMG, USA).

1.3.16. Biodistribution of Combo-NP *in vivo*

OCM1 cells were injected subcutaneously into the left flank of the BALB/c mice. Combo-NP@Cy7.5 was intravenously injected into the mice when the tumor volume reached 200 mm^3. An *IVIS* imaging system was used for fluorescence imaging at 1, 4, 7, 12, 24 and 48 h after injection. Finally, the tumor tissues and major organs (heart, liver, spleen, lung, kidney and intestine) were collected after 48 h injection and quantified using the *IVIS* Spectrum imaging system.

1.3.17. Antitumor efficacy evaluation in the OCM1 and B16F10 tumor model

To evaluate the antitumor effects of Combo-NP in a human-derived OCM1 tumor model and B16F10 tumor model, the BALB/c nude mice and male C57BL/6 subcutaneous tumor model were constructed by injecting OCM1 cells and B16F10 cells into the left flank of the mice, respectively. When the tumor volume reached approximately 80 mm^3, the mice were randomly divided into 5 groups (n = 5/group) and PBS, Len-NP, Combo-NP, PDT-NP+L, Combo-NP+L (equivalent to photosensitizer 10 mg/kg, Lenvatinib 8 mg/kg) were injected intravenously *via* the tail veins. The mice of laser groups were irradiated with a NIR light of 808 nm at intensity of 1 W/cm^2 for 3 min after 24 h injection. All the mice were treated every three days, and the tumor volume and body weight were monitored. The tumor volume was calculated as (length $\times$ $width^2$)/2. The mice were sacrificed after 15 or 16 days of treatments.

1.3.18. Immunohistochemical and immunofluorescence analyses

After completion of treatments, the mice were sacrificed and then the major organs and tumors were excised for histological observation by H&E staining and immunofluorescence (IF) staining. For H&E staining, excised tumors and organs were fixed in 4% paraformaldehyde

solution, embedded in paraffin sections, and stained with hematoxylin and eosin. Sections were then viewed under a fluorescent microscope (IX83, Olympus). TUNEL and Ki67 staining were used to assess apoptosis and proliferation of tumor tissues, respectively. The procedures were consistent with the manufacturer's protocol, and finally detected by CLSM.

1.3.19. Immunogenic cell death *in vivo*

To evaluate the release of HMGB1 and infiltration of $CD8^+$ T cells in tumor tissues, the mice were sacrificed and tumors tissues were separated, then the tumor tissues were fixed in 4% paraformaldehyde, processed, and embedded in paraffin. Paraffin tissue sections were stained with primary antibodies overnight at 4 °C. Secondary antibodies were added and incubated for 1 h. Nuclei were counterstained with DAPI and then the stained sections were imaged with a confocal microscope.

1.3.20. Tumor hypoxia and vascular normalization assays *in vivo*

To detect tumor hypoxia and tumor vascular normalization. The mice were sacrificed after complete treatments, and tumors tissues were fixed in 4% paraformaldehyde, processed, and embedded in paraffin. Paraffin tissue sections were stained with anti-Hif-1α to mark tumor hypoxia, followed by processing with second antibodies. Finally analyzed using a confocal microscope. Vascular normalization makers were labeled with anti-CD31 and anti-α-SAM antibodies, firstly, the tumor tissues were fixed, processed and embedded in paraffin, subsequent steps were consistent with the manufacturer's protocol, and finally detected by CLSM. The positive region of CD31 and α-SAM were analyzed by ImageJ.

1.3.21. Systemic antitumor mediated abscopal effect and metastatic tumor models

To evaluate the effects of the combination Combo-NP with α-PD-L1 mAb, B16F10 cells were injected into the left flank (primary tumor) of the C57BL/6 male mice. The mice were randomly grouped (n = 5 /group) including PBS, Len-NP, Combo-NP+L and Combo-NP+L+α-PD-L1 (equivalent to photosensitizer at 10 mg/kg, Lenvatinib at 8 mg/kg and α-PD-L1 at 10 mg/kg) when the tumor volume of primary tumor reached 80 mm^3. The mice of laser groups were irradiated to primary tumors, while the abscopal tumors were protected from irradiation after 24 h injection. All the mice were treated every three days and α-PD-L1 was injected intraperitoneally on days 1, 4, 7, 10. For the distant primary tumor models, the B16F10 cells were injected into the right flank (abscopal tumor) 4 days after establishment of the primary tumor model. Models of tumor metastasis were constructed by intravenously injecting B16F10

cells into the tail vein 4 days after establishing the primary tumor model. Finally, the mice were sacrificed and lungs were obtained.

1.3.22. Flow cytometry analysis of the animal tissues

The mice were sacrificed after treatments. The obtained fresh tumors, tumor-draining lymph modes and spleens were used to prepare single-cell suspension. Then the suspensions were further incubated with different antibody against the immune cells. To detect the DCs maturation in tumor and lymph modes, the cell suspensions were stained with antibodies of anti-CD11c-PE, anti-CD80-FITC and anti-CD86-APC, respectively. The matured DCs were marked as $CD11c^+CD80^+CD86^+$ cells. To detect the infiltration of antitumor T cells, the suspensions of tumor and spleen were incubated with anti-PE-CD3, anti-CD8-FITC and anti-CD4-APC and the $CD8^+$ T cells were denoted as $CD3^+CD4^-CD8^+$ cells. Finally, immunosuppressive cells including MDSC (characterized as $CD11b^+Gr\text{-}1^+$), Tregs ($CD4^+Foxp3^+$) and M2 (characterized as $F4/80^+$ $CD206^+$) in tumor tissues were also envaulted in the same way as above.

1.3.23. Statistical analysis

Experiments were performed at least three times and results were expressed as means ± SD. Statistical significances were analyzed using the one-way ANOVA test or two-way ANOVA test. The difference was regarded as significant when the *p* value was less than or equal to 0.05. $*p < 0.05$, $**p < 0.01$, $***p < 0.001$, $****p < 0.0001$, ns, not significant.

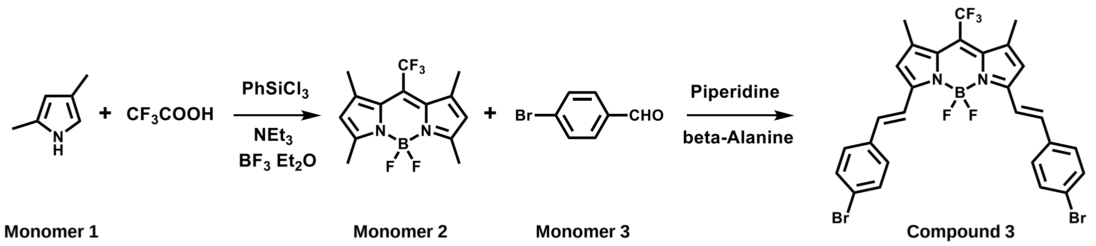

Figure S1. The synthetic route of Compound 3.

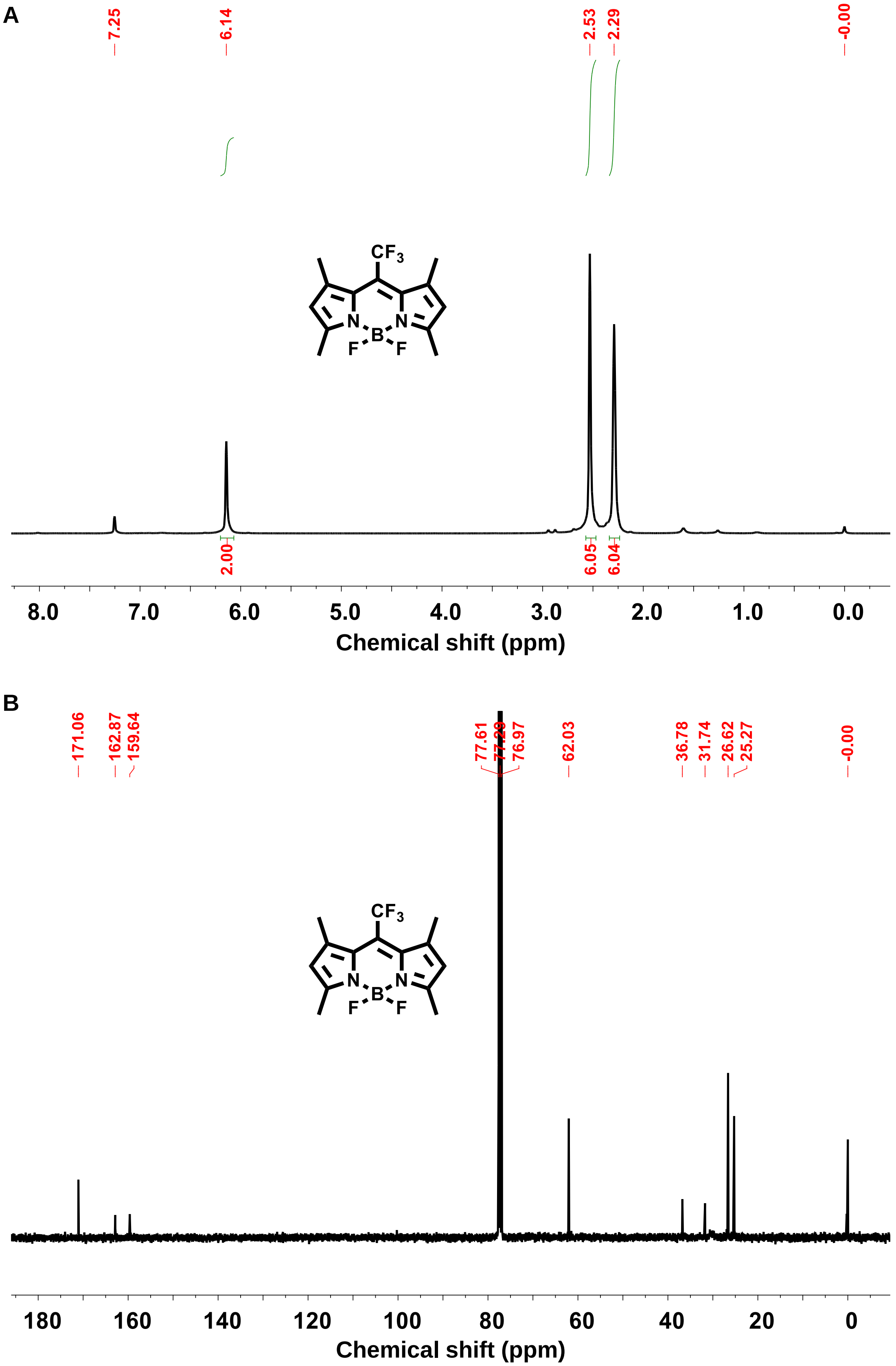

Figure S2. (A) ^{1}H NMR spectrum of Monomer 2 in $CDCl_3$. (B) ^{13}C NMR spectrum of Monomer 2 in $CDCl_3$. Copyright (2023), with permission from Elsevier.[2]

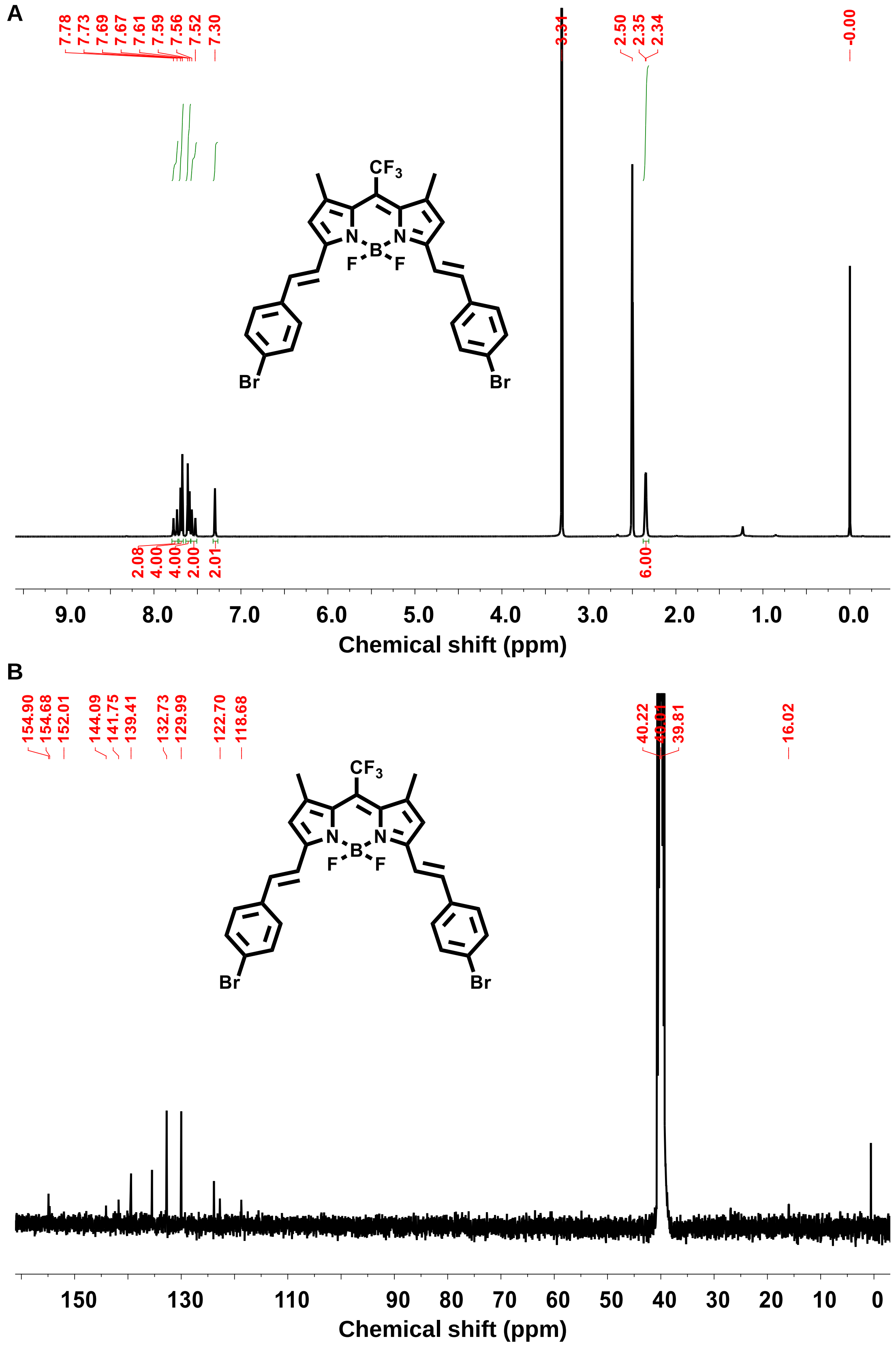

Figure S3. (A) ^{1}H NMR spectrum of Compound 3 in $CDCl_3$. (B) ^{13}C NMR spectrum of Compound 3 in $CDCl_3$. Copyright (2023), with permission from Elsevier.[2]

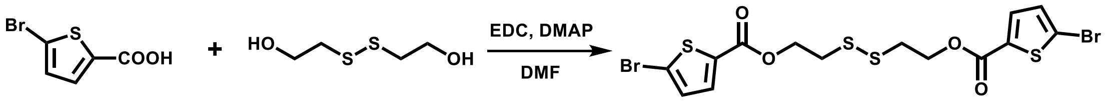

Monomer 4 Monomer 5 Compound 4

Figure S4. The synthetic route of Compound 4.

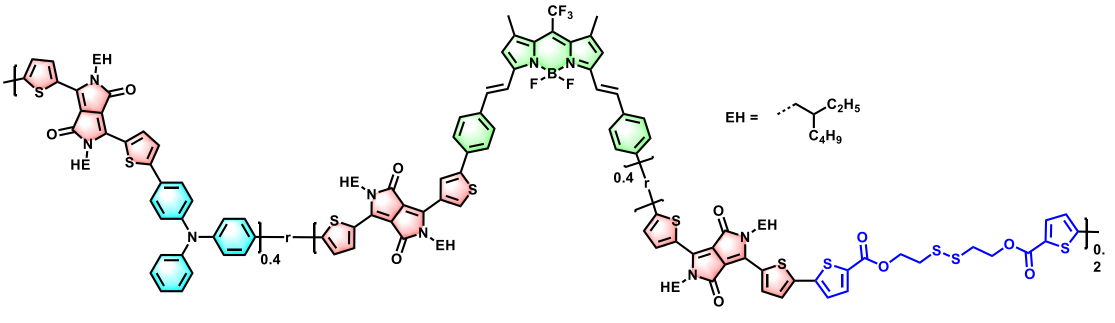

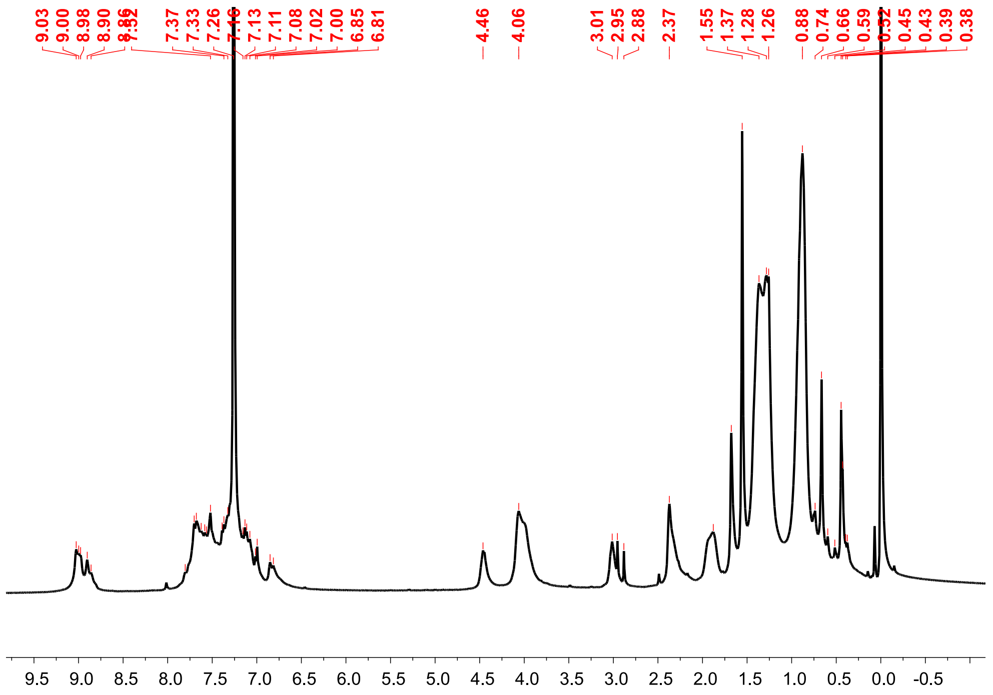

Figure S5. Characterization of TPA-BD by ^{1}H NMR.

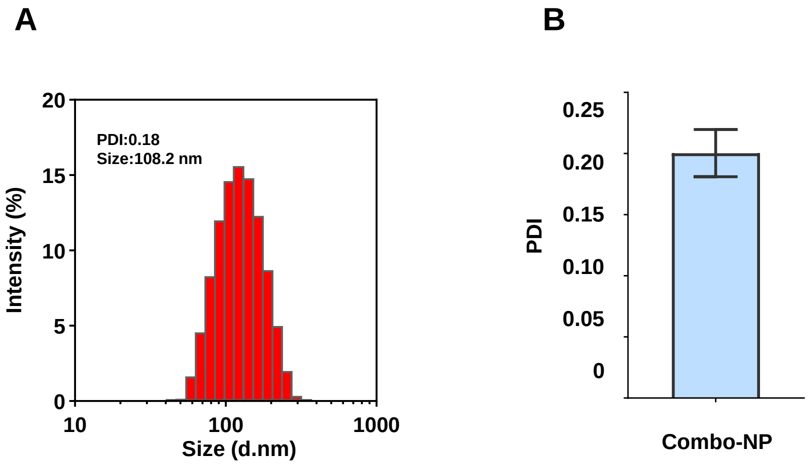

Figure S6. (A) Particle size of Combo-NP by DLS. (B) Particle dispersion coefficients of Combo-NP by DLS.

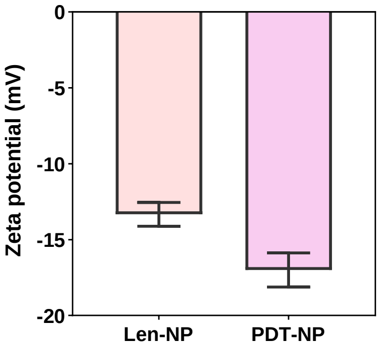

Figure S7. Zeta potential determined for the dispersion of Len-NP and PDT-NP.

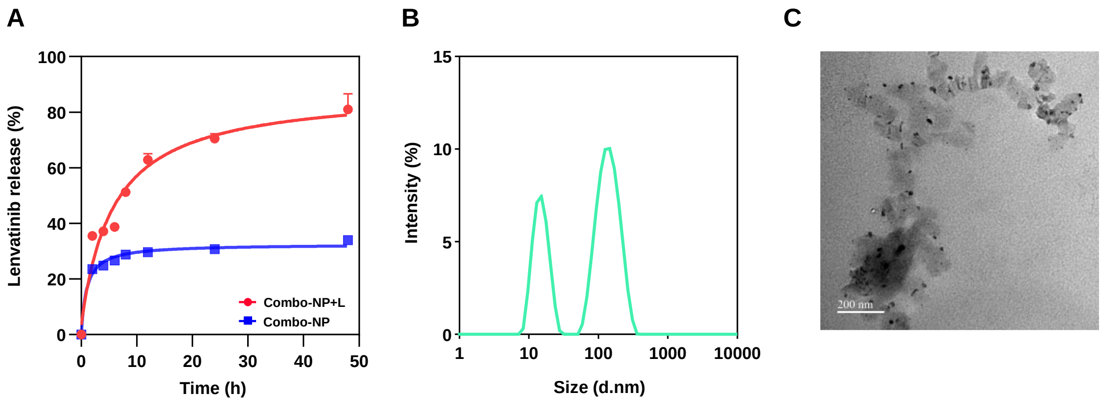

Figure S8. (A) The cumulative Lenvatinib release upon keeping of the sample in the dark or light irradiation. (B) The DLS characterization of Combo-NP after light irradiation. (C) The representative TEM image of Combo-NP after light irradiation. scale bar = 200 nm.

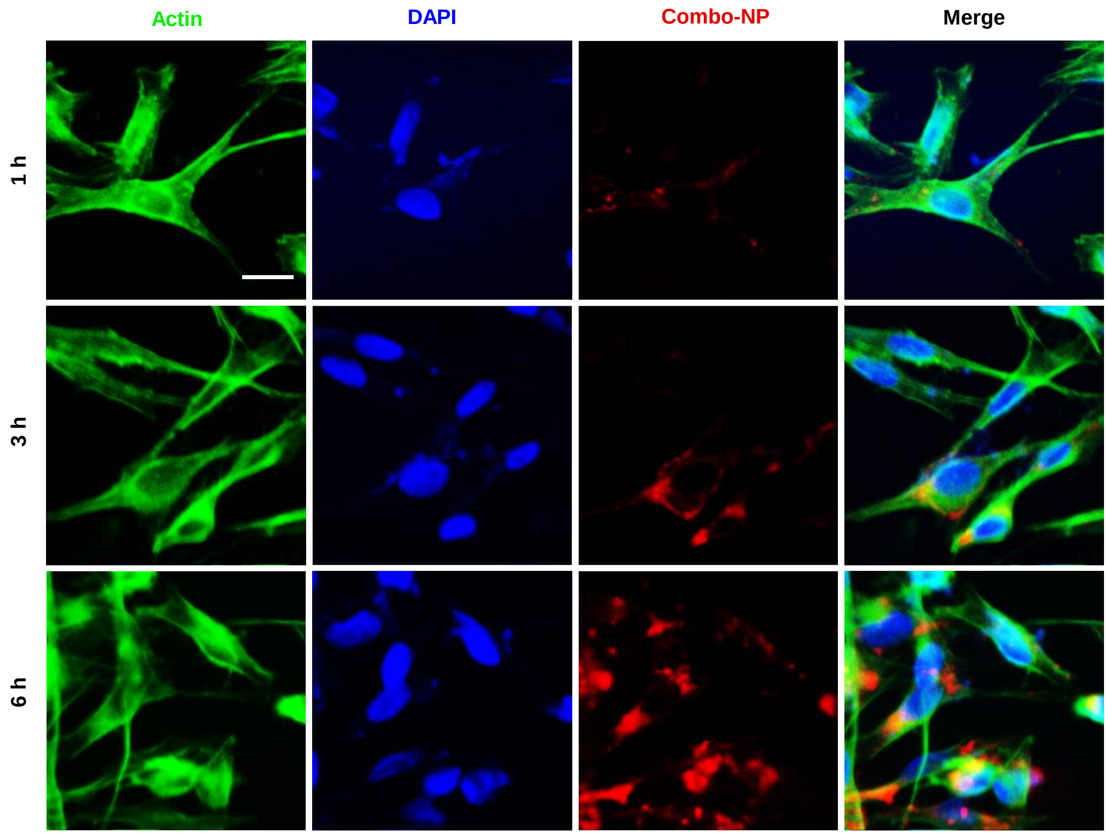

Figure S9. Intracellular uptake of Combo-NP in OCM1 cells by CLSM (NIR II, 808 nm) (3 h and 6 h have been shown in the body of the paper). Scale bar = 100 μm

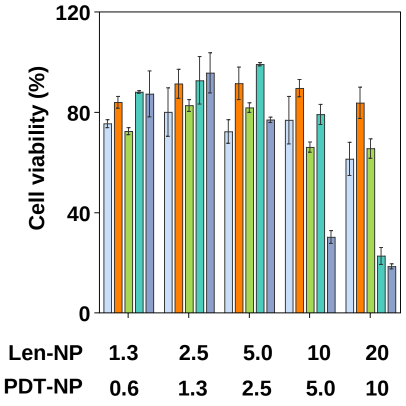

Figure S10. Cytotoxicity analysis of Len-NP (μM), PDT-NP (μg/mL), Combo-NP, PDT-NP+L and Combo-NP+L to B16F10 cells after 24 h by MTT.

A

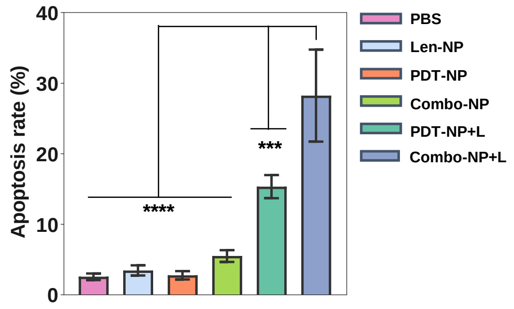

B

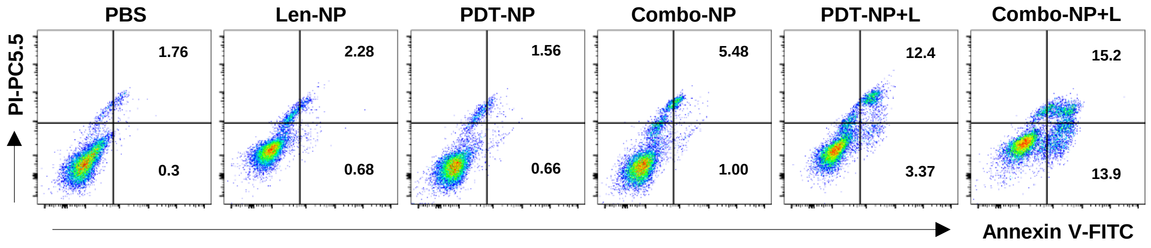

Figure S11. (A) The apoptosis rate in B16F10 cells and (B) the representative FCM images. n = 3. Data are presented as mean ± SD. Statistic significances between every two groups were calculated *via* one-way ANOVA. ***p<0.001, ****p<0.0001.

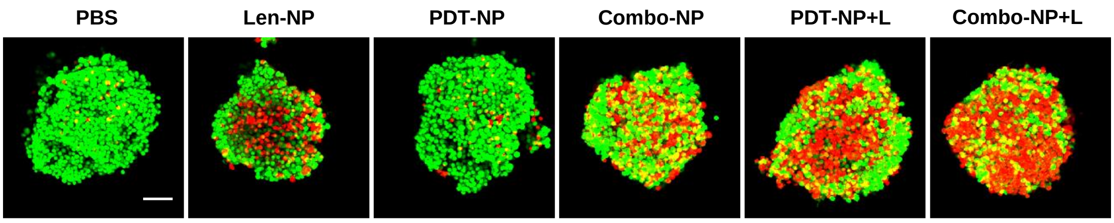

Figure S12. CLSM images of the 3D tumor spheroids of B16F10 cells stained with calcein-AM (green, viable) and PI (red, dead) treated with different drugs. Scale bar = 100 μm.

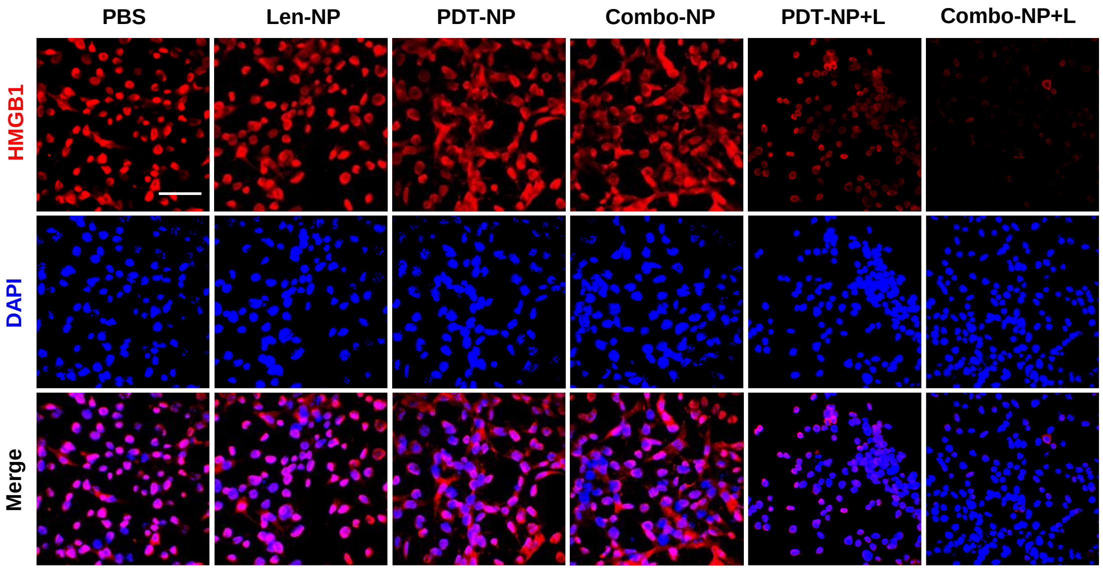

Figure S13. CLSM images of the release of HMGB1 in B16F10 cells after different treatments. Scale bar = 50 μm.

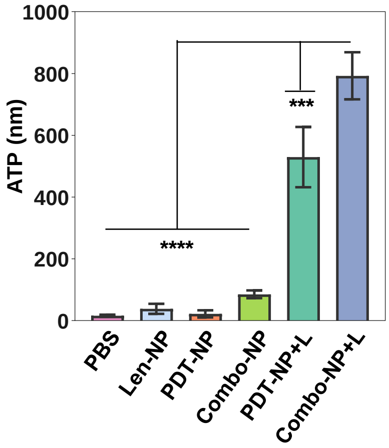

Figure S14. Extracellular ATP levels in B16F10 cells after various treatments. n = 3. Data are presented as mean ± SD. Statistical significances between every two groups were calculated via one-way ANOVA. * $p < 0.05$, **$p < 0.01$, *** $p < 0.001$, **** $p<0.0001$.

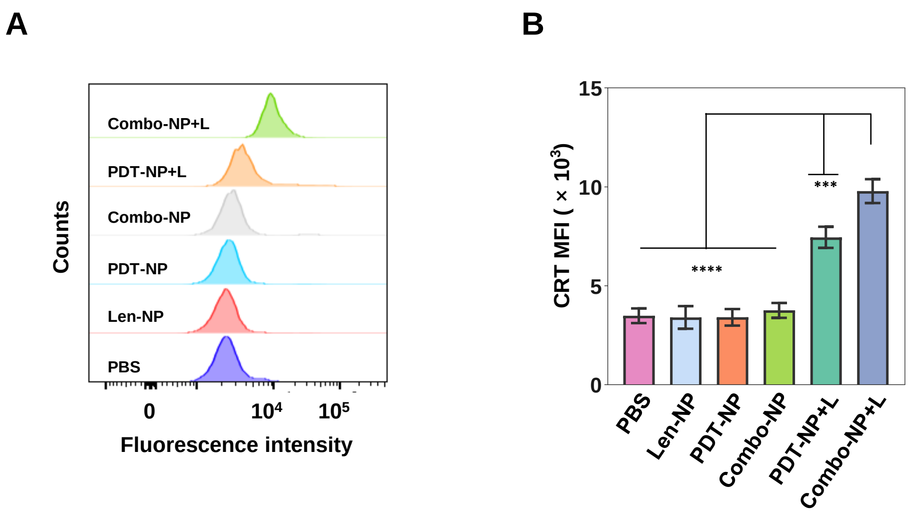

Figure S15. (A) Representative flow cytometric curves and (B) the corresponding quantification of surface expression of CRT on B16F10 cells. n = 3. Data are presented as mean ± SD. Statistical significances between every two groups were calculated via one-way ANOVA. * $p < 0.05$, ** $p < 0.01$, *** $p < 0.001$, **** $p<0.0001$.

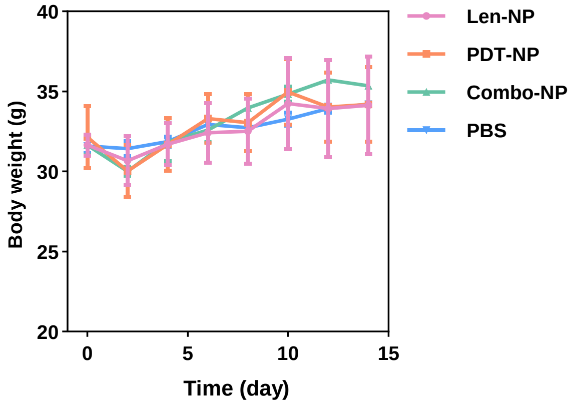

Figure S16. Body weight changes of KM mice after various treatments.

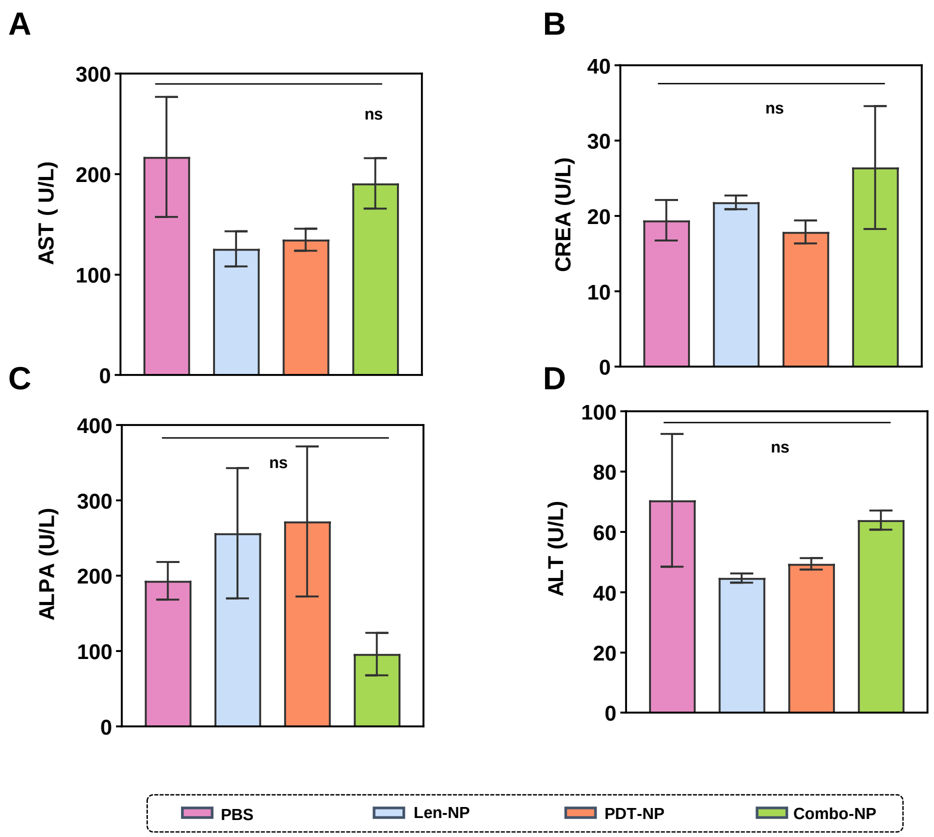

Figure S17. Biochemical analysis of serum: (A) aspartate aminotransferase (AST); (B) serum creatinine (CREA); (C) alkaline phosphatase (ALPA); (D) alanine aminotransferase (ALT). n = 3. Data are presented as mean ± SD. Statistical significances between every two groups were calculated via one-way ANOVA. * $p < 0.05$, **$p < 0.01$, *** $p < 0.001$, **** $p<0.0001$.

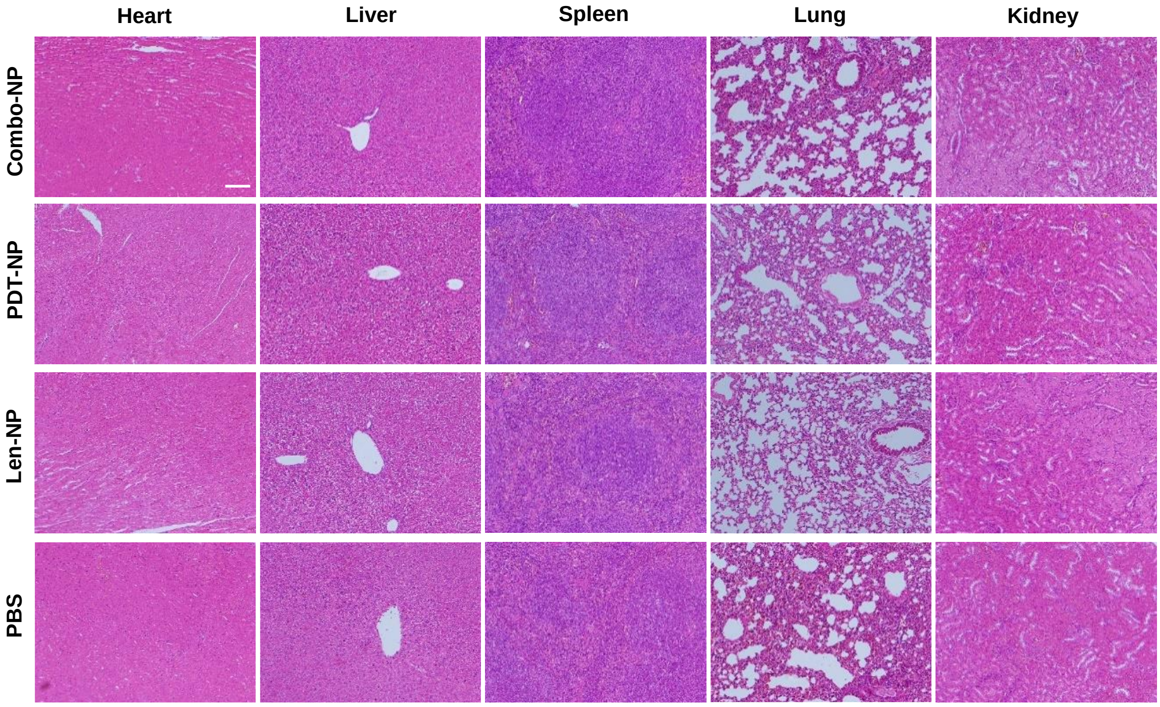

Figure S18. H&E staining of major organs (heart, liver, spleen, lung and kidney) after different treatments. Scale bar = 100 µm

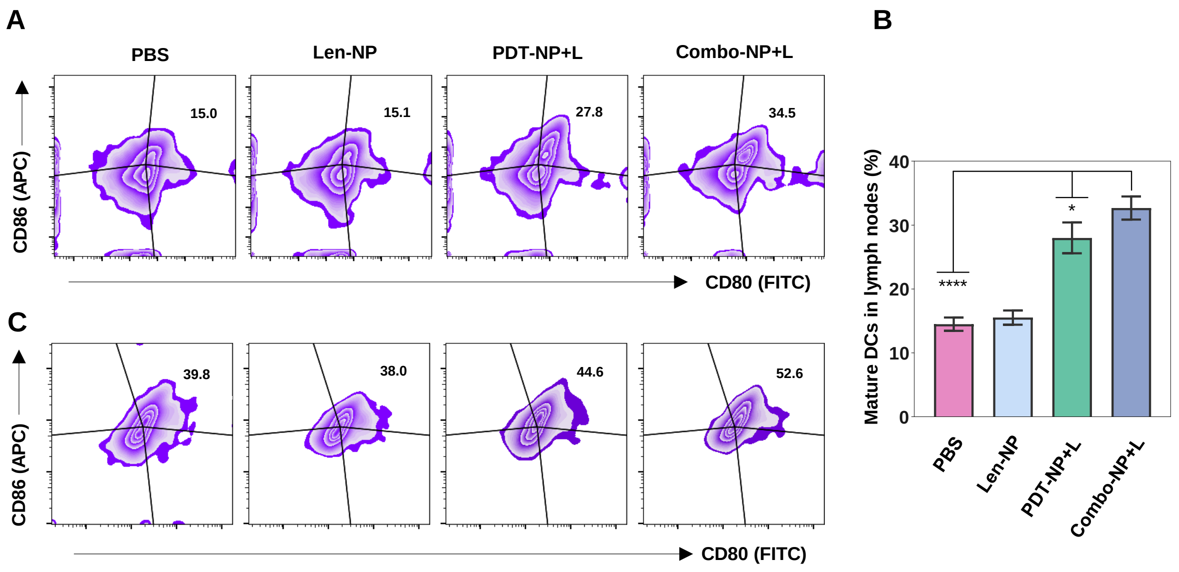

Figure S19. (A) Representative flow cytometric plots of matures DCs ($CD80^+$ $CD86^+$) and (B) percentages of DCs in lymph nodes. (C) Representative flow cytometric plots of matures DCs ($CD80^+$ $CD86^+$) in tumors. n = 3. Data are presented as mean ± SD. Statistical significances between every two groups were calculated via one-way ANOVA. * $p < 0.05$, ** $p < 0.01$, *** $p < 0.001$, **** $p < 0.0001$.

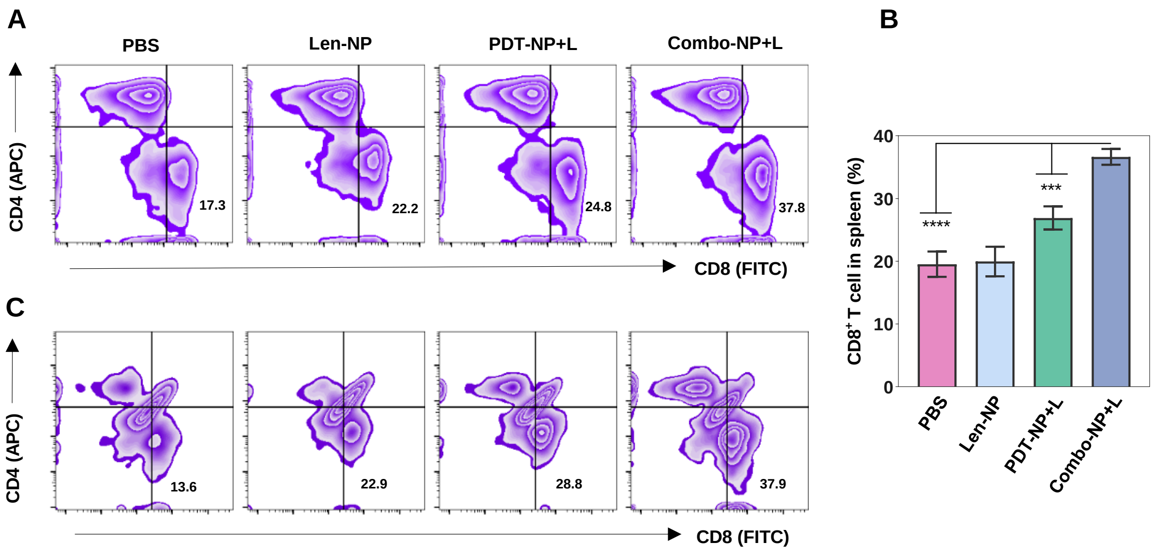

Figure S20. (A) Representative flow cytometric plots of $CD3^+CD8^+$T cells and (B) percentages of $CD3^+CD8^+$T cells in the spleen. (C) Representative flow cytometric plots of $CD3^+CD8^+$T cells in the tumor. n = 3. Data are presented as mean ± SD. Statistical significances between every two groups were calculated via one-way ANOVA. * $p < 0.05$, **$p < 0.01$, *** $p < 0.001$, **** $p<0.0001$.

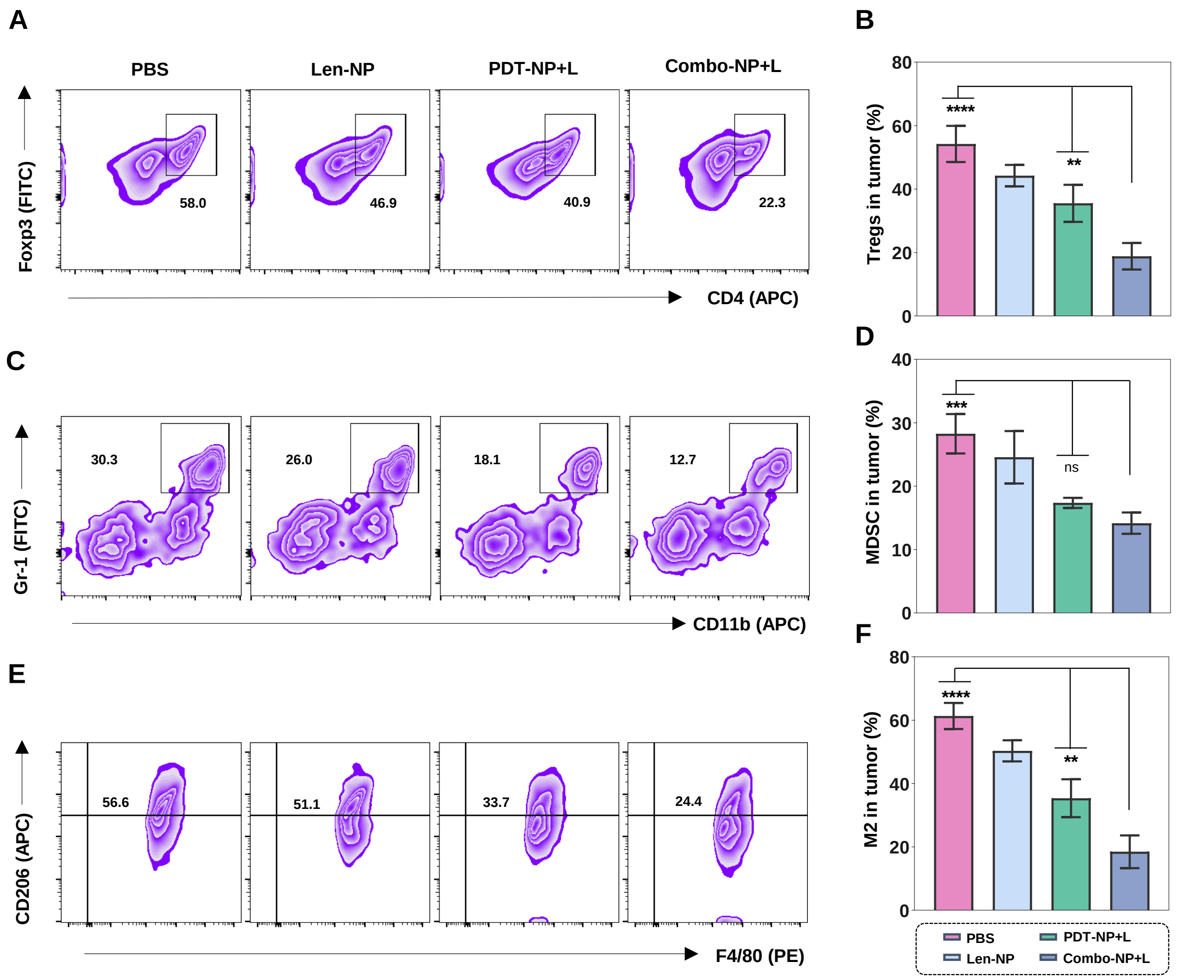

Figure S21. (A) Representative flow cytometric plots of Tregs (Foxp3^{+}CD4^{+}) and (B) percentages of Tregs in the tumor. (C) Representative flow cytometric plots of MDSC (Gr-1^{+}CD11b^{+}) and (D) percentages of MDSC in the tumor. (E) Representative flow cytometric plots of M2 (F4/80^{+} CD206^{+}) and (F) percentages of M2 in the tumor. n = 3. Data are presented as mean ± SD. Statistical significances between every two groups were calculated via one-way ANOVA. * $p < 0.05$, **$p < 0.01$, *** $p < 0.001$, **** $p<0.0001$.

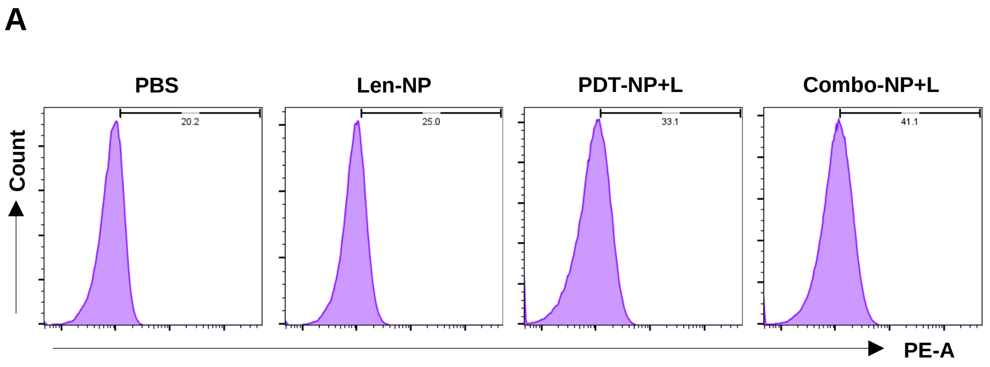

Figure S22. Representative flow cytometric plots of PD-L1 in the B16F10 tumor.

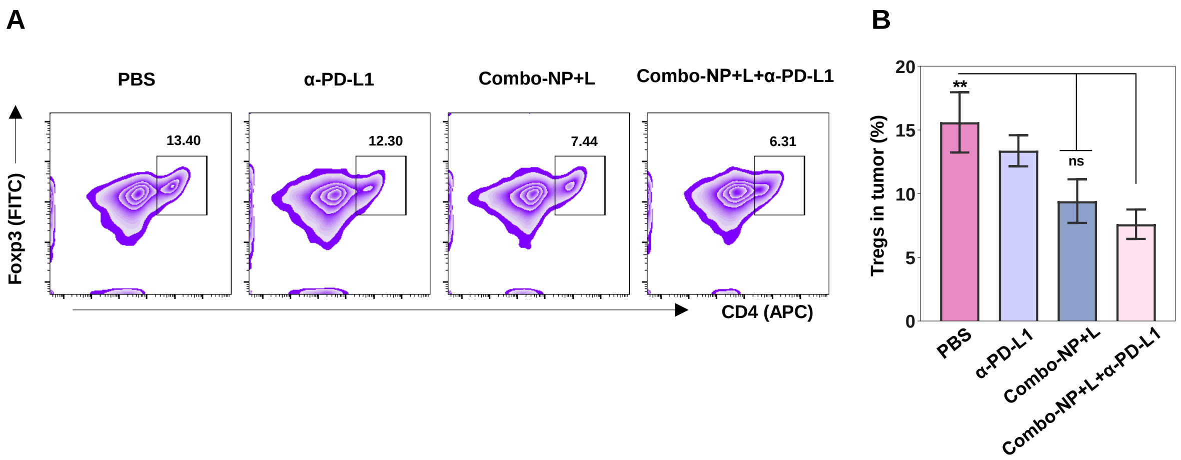

Figure S23. (A) Representative flow cytometric plots of Tregs ($Foxp3^+CD4^+$) and (B) percentages of Tregs in the distant tumor. n = 3. Data are presented as mean ± SD. Statistical significances between every two groups were calculated via one-way ANOVA. * $p < 0.05$, ** $p < 0.01$, *** $p < 0.001$, **** $p<0.0001$.

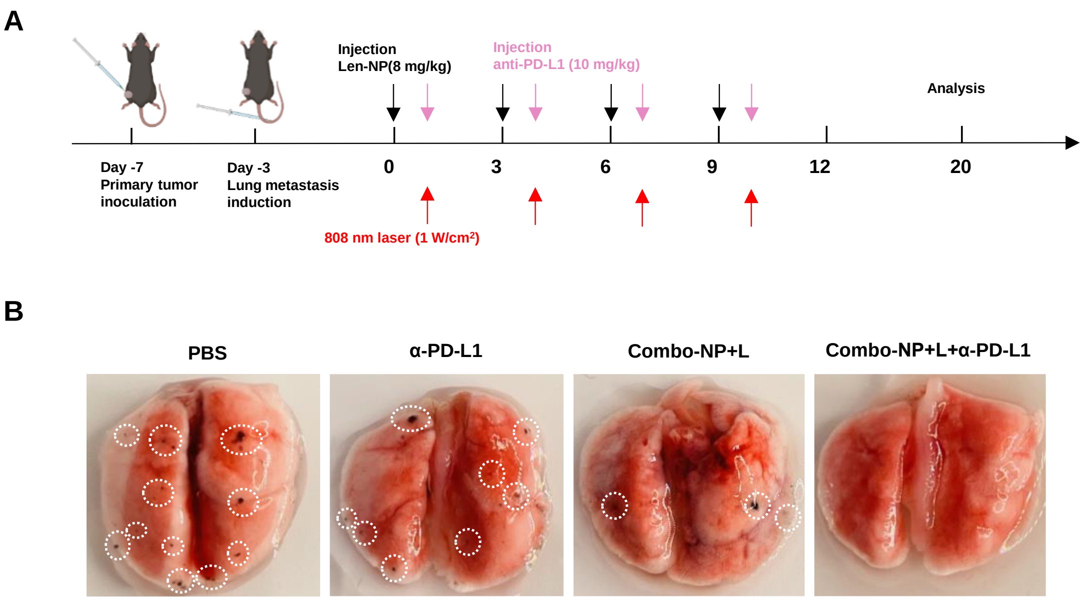

Figure S24. (A) Treatment schedule for in B16F10 metastasis models. (B) Observable metastatic nodules in the lung. Representative images of lungs in each group.

Table S1. Antibodies used for flow cytometry analysis

Antibody	Company	Catalog number
Goat polyclonal Secondary Antibody to Rabbit IgG-H&L (Alexa Fluor) ®488	abcam	ab150077
Goat polyclonal Secondary Antibody to Rabbit IgG-H&L (Alexa Fluor) ®555	abcam	ab150078
CD31	abcam	ab222783
α-SAM	Cell signaling	19245S
Hif-1α	abcam	ab179483
HMGB1	abcam	ab18256
CRT	Cell signaling	12238S
PE-CD3	elabscience	E-AB-F1013D
APC-CD4	elabscience	E-AB-F1097E
FITC-FOXP3	elabscience	E-AB-F1238C
FITC-CD8	elabscience	E-AB-F1104C
APC-CD206	elabscience	E-AB-F1135E
PE-F4/80	elabscience	E-AB-F0995D
PE-CD11c	elabscience	E-AB-F0991D
FITC-CD80	elabscience	E-AB-F0992C
APC-CD86	elabscience	E-AB-F0994E

References

[1] F. Ding, F. Li, D. Tang, B. Wang, J. Liu, X. Mao, J. Yin, H. Xiao, J. Wang, Z. Liu, Restoration of the Immunogenicity of Tumor Cells for Enhanced Cancer Therapy via Nanoparticle-Mediated Copper Chaperone Inhibition, Angew Chem Int Ed Engl 61(31) (2022) e202203546.

[2] X. Zhang, H. Hou, J. Wan, J. Yang, D. Tang, D. Zhao, T. Liu, K. Shang, *Nano Today* **2023**, *48*, 101759.

[3] Y. Yu, D. Tang, C. Liu, Q. Zhang, L. Tang, Y. Lu, H. Xiao, Adv Mater 2022, 34(4), e2105976.
